# Girls in STEM: Is It a Female Role-Model Thing?

**DOI:** 10.3389/fpsyg.2020.02204

**Published:** 2020-09-10

**Authors:** Susana González-Pérez, Ruth Mateos de Cabo, Milagros Sáinz

**Affiliations:** ^1^Department of Business Economics, School of Business & Economics, Universidad CEU San Pablo, Madrid, Spain; ^2^Internet Interdisciplinary Institute, Universitat Oberta de Catalunya (UOC), Barcelona, Spain

**Keywords:** gender role models, STEM, stereotypes, expectancy–value theory, career choice

## Abstract

Women are underrepresented in STEM (science, technology, engineering, and mathematics) careers, and this poses new challenges at the dawn of the era of digital transformation. The goal of the present study is to demonstrate how female role models influence girls’ preferences for STEM studies. This paper evaluates a role-model intervention in which female volunteers working in STEM go into schools to talk to girls about their careers. The study was conducted with 304 girls, from 12 years old (sixth primary grade) to 16 years old (fourth secondary grade), both before and after the role-model sessions. An adaptation of the expectancy–value theory of achievement motivation is used to test the extent to which this role-model intervention improves girls’ beliefs that they can be successful in STEM fields and increases their likelihood of choosing a STEM career. The results of multigroup structural equation modeling analysis show that on average, the role-model intervention has a positive and significant effect on mathematics enjoyment, importance attached to math, expectations of success in math, and girls’ aspirations in STEM, and a negative effect on gender stereotypes. Additionally, the female role-model sessions significantly increase the positive impact of expectations of success on STEM choices. Finally, the moderation role of the counterstereotypical content of the role-model sessions is tested. The results show that the higher the counterstereotypical character of the sessions, the higher the relationship between expectations of success in math and the choice of STEM. These results are discussed regarding their implications for long-term STEM engagement.

## Introduction

The proportion of women university students has increased from 46% in 1985 to 56% in 2017, and this has helped to improve their presence in the labor market, which on average means growth from 50.8% in 1985 to 52.5% in 2017 in countries belonging to the Organization for Economic Cooperation and Development (Organization for Economic Co-operation and Development (OECD), 2018a, b, c). However, gender equality in the workplace is far from being achieved. This labor gender gap is especially acute in professions that tend to be male dominated, with a high technological and mathematical component (i.e., fields known by the acronym STEM, for science, technology, engineering, and mathematics) (Kahn and Ginther, 2017). Indeed, women in OECD countries account for only 19% of entrants into tertiary level in these programs (Organization for Economic Co-operation and Development (OECD), 2018c).

Spain provides a paradigmatic picture of this situation. Despite of being reported as one of the countries with greater improvement in the most-recent edition of the Global Gender Gap Report (entering the top 10 from the previous edition’s 29th position out of 153 counties in 2019), establishing itself as a champion against gender discrimination (World Economic Forum [WEF], 2020), large gender gaps in wages remain, income, and the presence of women in managerial positions. The labor participation of women is also lagging behind that of men (68.8% versus 78.9%). Advances already achieved are now in jeopardy with the digital transformation of the labor market, which might increase the economic gender gap produced by the underrepresentation of women in emerging professions. In Spain (Ministerio de Educación y Formación Profesional (MEFP), 2019), women are severely underrepresented in physical science (25.3%), electrical engineering (20.5%), electronics engineering (15.2%), computer science (12.0%), civil engineering (28.3%), industrial engineering (24.7%) and aeronautical engineering (23.5%), and they are overrepresented in fields oriented to biology and health, such as medicine (66.4%), biomedical engineering (59.1%), biology (61.8%), and chemistry (54.2%). The proportion of women with degrees in mathematics is even lower than it used to be (36.6% in 2019–2020 vs. 39.0% in 2015–2016) (Ministerio de Educación y Formación Profesional (MEFP), 2020). Removing the barriers that prevent women from accessing the science, research, and technology sectors will be key to changing the current academic orientation, which is essential for combating new forms of gender inequality (Shapiro et al., 2011).

This pattern of low representation of women in the STEM disciplines can also be seen in many Western and European countries. Indeed, the lack of girls choosing scientific studies may mean there is no critical mass of candidates prepared to access leadership positions (Kanter, 1977) and result in the exclusion of the feminine perspective in creating and developing solutions (World Economic Forum [WEF], 2020). Moreover, women should not miss out on fulfilling, rewarding, and highly paid careers in STEM, where employment growth rate is three times faster than for non-STEM jobs (Langdon et al., 2011). Educational background is also increasingly important in the appointment of directors to boards (Hitt and Tyler, 1991), where technological profiles are in high demand (Ruigrok et al., 2007) because they drive research and innovation (Barker and Mueller, 2002).

Much research has been devoted to identifying the beliefs of students about STEM competences and gendered motivational factors that influence their educational and career decisions (Hackett and Betz, 1981; Quimby and O’Brien, 2004; Sáinz and Eccles, 2012; Watt et al., 2012; Wang and Degol, 2014; Wang et al., 2017). This research draws on the expectancy–value theory of achievement motivation by Eccles and Wigfield (1995) and Wigfield and Eccles (2000). According to this theory (Eccles et al., 1983; Eccles, 2005), when expectations of success and the value of STEM degrees and careers are high, girls are much more likely to choose STEM pathways. Existing stereotypes about the nature of STEM work and people working in STEM become powerful drivers of gendered aspirations and affinities (Thébaud and Charles, 2018; Sáinz et al., 2019), supporting women’s STEM avoidance and men’s STEM affinity (Glick and Fiske, 1999; Diekman and Eagly, 2008). A good way of overcoming stereotype barriers is through the intervention of female role models, who can increase the sense of belonging to STEM and reinforce the idea that hard work is the way to succeed in STEM (Weisgram and Bigler, 2007; Shin et al., 2016; Bertrand and Duflo, 2017).

In this study, we examine the effectiveness of a current and innovative role-model-based intervention on the perceptions that young girls (from 12 to 16 years old) have of gender stereotypes about mathematical competence, expectation of success in math, their degree of math enjoyment, and the importance attached to math, and how all of these contribute to shaping the likelihood that girls will choose STEM careers. We expect that the exposure to successful female role models in STEM fields could serve as a key driver to convey that they can succeed in these careers while still having a personal life (Marx et al., 2005; Williams and Ceci, 2012; Sáinz et al., 2019). This is especially relevant during the first stages of education because there is a consensus that the progressive abandonment of girls in some STEM fields (the start of the “leaking pipeline”) begins after the age of 12 (Sáinz and Eccles, 2012) given the predisposition of girls to underestimate their ability to be successful in STEM fields (Correll, 2001; Sáinz and Eccles, 2012).

The present study is especially innovative because it analyzes a field intervention involving actual female role models for young girls in schools at a national level. This is important because the relatively few existing studies on the impact of role models on the intention to pursue STEM careers (Plant et al., 2009; Stout et al., 2011; Van Camp et al., 2019) use mainly: computer-based agents, STEM role-model biographies, exposure to upper-level undergraduates, female professors, or female peer experts for female students who are already majoring in STEM disciplines, which can limit the scope and validity of their results. However, our study is carried out with actual STEM role-model women who are physically present in the classroom and who are talking in first-person terms about their own lives and professional experiences to young girls at a decisive stage of their lives (preadolescence), which is precisely when they start to consider dropping out from these disciplines because their individual self-efficacy is in flux. We consider that in comparison with other experimental designs, the present context could improve the closeness and experience that female role models provide to the young girls and, as a consequence, could boost the potential impact that this type of intervention has on their final intention to pursue STEM careers.

## Theory and Hypotheses

### STEM Career Choice: Expectancy–Value Theory

The expectancy–value theory developed by Eccles and her colleagues proposes that achievement-related choices can be predicted by the expectations a person has of succeeding, as well as subjective task values (Eccles et al., 1983; Eccles, 2005). This model has been used in different fields (e.g., math, reading, computing, health, communications, sports, marketing, and economics) and specifically when trying to explain the gender gaps in STEM (e.g., Sáinz and Eccles, 2012; Eccles, 2015). The expectations of success and subjective task values are presumed to directly influence achievement-related choices, performance, and persistence (Eccles, 2015). Students will, therefore, be more likely to pursue those studies and academic options in which they think they can excel or that have a high value for them (Eccles and Wigfield, 1995; Sáinz and Eccles, 2012). That is, when expectations of success and the value of STEM disciplines are high, girls are much more likely to choose, persist in, and graduate from STEM fields (Eccles and Wigfield, 1995; Sáinz and Eccles, 2012; Eccles, 2015).

Expectancies and values are the two main components of the model, which, although different constructs, are highly correlated. Expectancies of success tend to predict children’s task values. Whereas subjective task values are closely linked to educational or career choices (Wigfield and Eccles, 2002; Durik et al., 2006; Eccles, 2009; Wang et al., 2015), expectancies of success (i.e., self-concept of ability or self-perception of competence) are strongly related to performance.

According to the theory (Wigfield and Eccles, 2002; Eccles, 2005; Wigfield et al., 2006), expectations of success and task values are shaped by a combination of factors, from individual child characteristics (e.g., abilities, previous experiences, goals, self-concepts, beliefs, and expectations) to environmental influences (e.g., cultural milieu, peer beliefs, and behaviors). Subjective task values include the following motivational factors: attainment value or importance, intrinsic value (enjoyment), utility or usefulness of the task, and costs [Eccles et al. (1983) and Wigfield and Eccles (1992) discuss these components in more detail].

The influences of family, school, peers, mass media, and the immediate social environment shape the expectations that girls and boys have of success (and their self-concept of their own abilities) together with the value they attach to various subjects and academic domains (Eccles, 1994). Encouragement received from significant people (family, schools, peers, and others) to pursue mathematics or technology-related studies plays a major role in whether or not adolescents decide to pursue a career in STEM domains (Sáinz and Eccles, 2012; Eccles, 2015).

**FIGURE 1:**
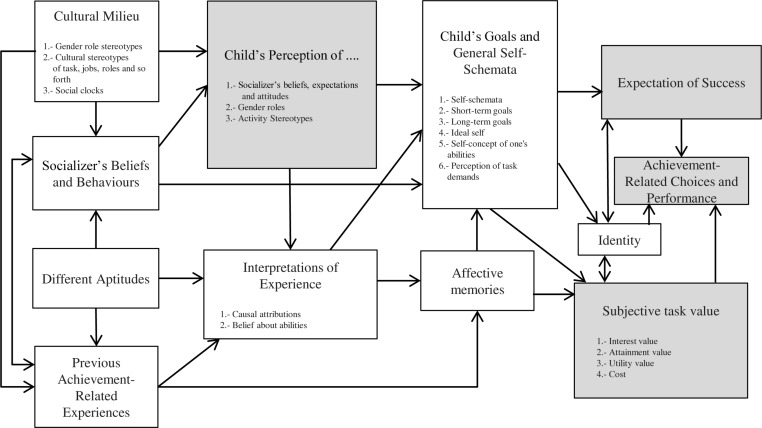
Selected constructs (shaded boxes) of the Eccles expectancy-value model of achievement-related choices.

Shin et al. (2016) identified two stereotypes that affect the level of recruitment and retention of women in STEM fields. On one hand, there is the idea that STEM studies are difficult, and a person should be a brilliant or gifted student to succeed in them. On the other hand, there are cultural and social stereotypes about the characteristics of scientists and scientific jobs (i.e., people lacking social abilities, with an unattractive physical appearance, or freaks) that undermine the interest that girls may have in STEM, as they do not match these stereotypes. Further empirical research supports this analytical view (Cheryan et al., 2015; Sáinz et al., 2016, 2019). Shin et al. (2016) agree that a good way of overcoming these two barriers is through the intervention of female role models, as they can increase the sense of belonging to STEM and reinforce the idea that hard work is the way to succeed in STEM. We present the overall model to provide a sense of its scope (Figure 1).

The present research focuses on how two elements of the task values (personal enjoyment and the importance attached to math) along with expectations of success predict the future STEM aspirations of a group of girls before and after having participated in a female role-model intervention. For this purpose, we focus the present work on a portion of the model; specifically, the constructs contained in the boxes related to expectancies and subjective task values boxes, along with the construct of child perception of gender role stereotypes (shaded boxes in Figure 1).

Expectations of success depend on both the confidence that individuals have in their various intellectual abilities, on their estimations of the difficulty of the options they are considering and on their estimates of the external or societal barriers to their success (Eccles, 1987; Eccles, 2005). Regarding the subjective task values, according to the theory (Eccles et al., 1983; Wigfield and Eccles, 1992; Eccles, 2005), they are assigned to a task (e.g., math) based on interest or personal enjoyment (intrinsic value), utility value, and attainment value. More specifically, interest or intrinsic value is the enjoyment one gains from doing the task (i.e., in our case the enjoyment value of doing a math exercise); attainment value is defined as the importance of doing well at a given task, which is given by the link between the mathematical topic and one’s sense of self and identity; and utility value or usefulness refers to how a task fits into an individual’s future plans, for instance, taking a math class to fulfill a requirement for a science degree. The latter two are usually combined and known as the importance value (e.g., Wigfield and Eccles, 2002; Eccles, 2005), so we have grouped them in that way. As we can see in our theoretical model, although being highly correlated, all these motivational factors tends to predict the choice of a STEM career in a positive way.

The theory also considers the role played by gender stereotypes (another social–cognitive process) in shaping gender differences in the choice of a STEM career (Bussey and Bandura, 1999). Girls tend to move away from some STEM disciplines, as success in a STEM career is commonly associated with a high degree of intellectual brilliance, and brilliantness is stereotypically correlated with masculinity (Eccles et al., 1998; Guimond and Roussel, 2001; Sáinz et al., 2019). Frenzel et al. (2007) found that girls, when addressing a scientific problem, were more insecure and considered themselves more incompetent, and that their degree of enjoyment was lower. This is why women tend to avoid disciplines with a strong mathematical load (Wigfield and Eccles, 2002; Eccles, 2005, 2008; Sáinz and Eccles, 2012). In this way, gender-role stereotypes in math should act as a direct deterrent when it comes to choosing a STEM career.

**FIGURE 2:**
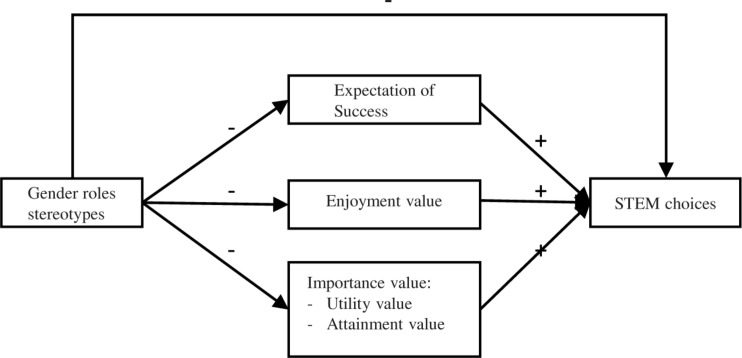
Theoretical STEM-choice model.

As illustrated in Figure 2, the present paper theorizes that a girl’s choice of a STEM career can be explained by the relationships among the following key identity, social, and motivational factors associated with math (a basic required competence across STEM fields that is the basis of science higher education in most academic institutions and that students often have to choose as they advance academically): expectations of success, math enjoyment, importance, and gender stereotypes about math ability.

All that said, for a better understanding of how gender-role stereotypes and motivational factors prevent or encourage girls from entering STEM careers, we propose the following hypothesis:

H1: Expectancy–value-related motivational factors predict positively female-student preferences for a STEM career, whereas gender-role stereotypes have a negative effect both in these motivational factors and on the intention to pursue a STEM career.

### Role-Model Influence

Role models can be inspirational and can reduce the self-stereotyping of stigmatized groups, and this may be the case for women in male-dominated STEM fields (Lockwood, 2006; Betz and Sekaquaptewa, 2012; O’Brien et al., 2017). Interventions based on role models revolve around enhancing a sense of belonging and identity in STEM fields, thereby fostering the personal connections of girls to the STEM community (Casad et al., 2018; Van Camp et al., 2019). Scholars have identified two factors that affect the efficacy of the inspiration that role models provide for individuals (Lockwood and Kunda, 1997), namely (i) the perceived relevance of the role model to the individual (i.e., domain relevance) and (ii) the believed attainability of the role model’s success. Dasgupta (2011) used the theoretical lens of the stereotype inoculation model to explain how contact with successful female STEM role models can serve as “social vaccines” that protect the self-concept of women in STEM against stereotypes.

There is evidence that among STEM women, perceptions of incompatibility between their gender and STEM identities (i.e., the extent to which people perceive their identity as a woman or man to fit with their identity as a STEM member) are related to a lesser sense of belonging, greater insecurity, and less motivation in STEM, as well as greater expectations of dropping out of STEM (London et al., 2011). Same-gender role models seem to be a more effective option for attracting young women into STEM (Bussey and Bandura, 1999; Cheryan et al., 2011; Stout et al., 2011). Female-oriented STEM promotion thus requires role models (sometimes mentors) who not only work in a STEM field but who are also female. However, because the participation rates of women in these fields are low, finding a sufficient number of professional women in STEM fields such as engineering and physical science is challenging. This could explain the sparse research about the influence of same-sex role models on the intention to choose a STEM career and the use of computer-based agents, biographies, or teachers as close substitutes for actual female STEM role models. So, Stout et al. (2011) discovered that women exposed to female calculus professors showed enhanced self-efficacy, greater self-concept, as well as a higher identification with and commitment to STEM, even among students who still maintained gender stereotypes. In a similar vein, Plant et al. (2009) exposed middle-school girls to computer-based female role models and found that the role model was effective at promoting academic interest and motivation among girls. It is therefore not surprising that exposure to role-model biographies that challenge common STEM stereotypes (e.g., that STEM is for gifted individuals) has also been demonstrated to have positive effects on both STEM and non-STEM student interests in STEM, as well as their perceived identity compatibility between themselves and STEM (Shin et al., 2016; Sáinz et al., 2019). Role-model exposure also has a positive impact on academic sense of belonging among STEM and non-STEM students, and a positive impact on academic self-efficacy among STEM students, but not non-STEM students (Shin et al., 2016).

Numerous and varied initiatives based on role models have been launched all over the world to make STEM fields more attractive to girls and increase their interest in these professions (Van Camp et al., 2019; Sáinz, 2020). As in the case of any other intervention, the effectiveness of the female role-model-oriented intervention depends on several indicators, such as the scope of the intervention, the theoretical background inspiring it, the design, measures, and tools to evaluate its impact, educational agents involved in the intervention, its sustainability, and so on (Sáinz, 2020). For instance, Breda et al. (2018) demonstrated that girls participating in the intervention had a lower level of stereotypes than did girls in the control group. Their STEM interest also increased by between 20 and 30% above that of the control group. Equally, the probability that top-performing girls in math would be enrolled in a STEM program increased by 50% from a baseline of 28%. Among these top-achieving girls, the program reduced the baseline gender gap in the enrolment in selective STEM programs by a third, from 22 to 14%. In comparison to this previous research, our study contributes to literature analyzing the influence of a two-step female role-model intervention not only on female students’ gender stereotypes about women’s STEM abilities, but also on female students’ motivational outcomes (i.e., expectation of success, enjoyment, and importance value), as well as, interest in pursuing STEM fields. Additionally, we look at changes in not only mean values but also in the relationships among the model variables.

Indeed, not only do role models and mentors help broaden the perspectives of who can work in the STEM field, they also expand students’ perceptions of their own potential (Johnson et al., 2020). Research shows the positive impact of interaction with STEM professionals on students’ STEM interest (Kesar, 2018). Therefore, it is reasonable to expect that girls are more motivated (in terms of expectation of success, enjoyment, and importance) to engage in subjects related with STEM fields, such as math, after interacting with female role models in STEM than before doing so.

Based on the above rationale, to evaluate the effectiveness of the female role models’ intervention, we posit the following hypotheses:

H2a: Female-student participation in the role-model sessions increases the mean value of the motivational factors considered in our theoretical model (expectations of success, enjoyment, and importance), as well as that of the intention to pursue a STEM career, while decreasing the mean value of gender stereotypes about STEM abilities.

H2b: Female-student participation in the role-model sessions increases the positive effect of the motivational factors (expectations of success, enjoyment, and importance) on the intention to pursue a STEM career, while strengthening the negative impact of gender stereotypes on these motivational factors, as well as on preferences for a STEM career (i.e., role-model sessions have a moderator effect).

### Counterstereotypical Role-Model Influence on Girls’ STEM Choices

According to gender theories [gender schema theory (Martin and Halverson, 1981) and social role theory (Eagly and Wood, 2011)], people perceive certain roles to be more or less appropriate for their gender. This means that observing men and women in gender-congruent roles fosters gender-congruent aspirations and behavior. By contrast, following the rationale that observing or interacting with men and women in non-traditional domains provides a so-called gender-counterstereotypical role model, the frequent exposure to gender-incongruent role models should reduce gender stereotyping and promote non-traditional behavior (Olsson and Martiny, 2018).

Based on this idea, numerous initiatives and research-based interventions involving observing or interacting with gender-counterstereotypical role models have been implemented in several countries, particularly focusing on outcomes for girls and women. The review of these interventions carried out by Olsson and Martiny (2018) shows that exposure to or interaction with counterstereotypical role models can reduce gender stereotyping during childhood and preadolescence among girls on a short-term basis. However, the changes in stereotypes are not always sustained and do not necessarily affect children’s aspirations and behavior. In this regard, Olsson and Martiny (2018) recommend that future research should assess whether a change in stereotypes is internalized and acted upon. The present work follows this recommendation by investigating not only the changes in young girls’ gender stereotypes after the intervention but also its influence on their STEM career aspirations.

Although STEM stereotypes are incongruent with the female gender role, they can be conveyed to a certain degree by women as well (Cheryan et al., 2011). In STEM, these stereotypes include a tendency toward social isolation and a singular focus on technology (Barbercheck, 2001). In contrast, the female gender role prescribes many opposing characteristics, such as helping and working with others (e.g., teamwork) and being socially skilled (e.g., communication skills). Young girls who encounter stereotypical STEM role models may feel dissimilar from them and, as a result, the talks of those stereotypical role models who are supposed to inspire emulation may have a lower effect or even deter those they were meant to benefit (Cheryan et al., 2011). For this reason, when we look for a specific measure of the counterstereotypical content of the female role-model sessions, we focus on the congruent content that the girls may be provided with by the female role models during the sessions. A content is defined as congruent if the role-model discourse includes, among the requirements to follow a STEM profession, the demand for non-stereotypical skills (especially, social and communications skills, which are congruent with their gender behavior).

Against this background, we tested for differences in our model relations within the post-intervention sample between those sessions perceived as highly counterstereotypical by the girls and those that were not. To test this, we postulate our third hypothesis:

H3: Highly counterstereotypical role-model sessions strengthen the possible changes observed in the relationship(s) of the constructs in the theoretical model (i.e., the counterstereotypical content of the sessions plays a role as a strength moderator).

## Materials and Methods

### Procedure

This study is part of the program of the Inspiring Girls Foundation (IGF), whose main objective is to raise girls’ aspirations in STEM by connecting them with female role models. The IGF has implemented a cutting-edge program, recruiting top women leaders from STEM companies as role models to go into schools to talk about their careers and experiences in the profession. All volunteers follow an innovative digital onboarding training process before engaging in the program. This training highlights the importance of volunteers talking about the opportunities and requirements to enter their jobs, the contribution that their jobs make to the real world, and the opportunities for making work and private life compatible, as well as the negative effects of gender stereotypes in career choices. The sessions are organized through a platform for role models, where participating schools can access female STEM experts directly (Inspiring Girls Foundation [IGF], 2018). Another key strength of this project is that each group of students meets three female role models. These interactions increase the probability that girls are exposed to women with diverse personality traits, physical appearance, socio-demographical characteristics (e.g., civil status and number of children), ages, and professional paths, which provides the intervention with a higher diversity and inclusiveness compared with other experimental designs.

### Sample

We designed two questionnaires that were administered in 2018–2019 to 304 girls from 12 years old (sixth primary grade) to 16 years old (fourth secondary grade) who responded before and after the role-model sessions. The first questionnaire was administered 1 week before the role-model session and the second 1 month after. A total of 16 schools participated: 11 were public and five were private. At nine schools, the family income of the students was the average for Spain; at the other seven, it was above average. Seven schools were in the south of Spain (Malaga, Seville, Almeria, Cadiz, and Huelva), four in the center (Madrid and Toledo), four in the east (Barcelona, Valencia, and Alicante), and one in the north (Navarra).

### Study Design

The empirical strategy was as follows. First, the relationship between the social (stereotypes) and motivational factors in our theoretical STEM-choice model were tested by using structural equation modeling (SEM) with the whole data set (H1). Next, the effectiveness of female role-model interventions was examined, comparing the differences in the mean of the constructs (H2a) and changes in the relationships after the role-model sessions (H2b). Finally, we tested for differences in these relationships between the sessions perceived as highly counterstereotypical by the girls and those that were not (H3). To do this, we ran a multigroup SEM within the post-intervention sample. Within this post-intervention sample, we tested for invariance in the changed relationships after the intervention between (i) the sessions considered highly counterstereotypical by the girls in terms of the content that they were provided with and (ii) those that were not, to check for the possible strength-moderator effect of this characteristic of the sessions.

For ethical reasons, the IGF considers it inappropriate to assign schoolchildren randomly to a particular female role model. Therefore, the present research used a one-group pre-test and post-test design methodology (Campbell and Stanley, 1963). Pre-test/post-test designs are used widely in behavioral research, primarily to compare groups and/or measure changes resulting from experimental treatments and interventions (Dimitrov and Rumrill, 2003). To avoid the risks related to the internal validity of this type of design (Knapp, 2016), and to minimize the negative effects that could stem from the absence of a control group, we applied the following rules. To reduce the effects of history (i.e., some other event occurring at the same time of the intervention that could be the cause of the change in the outcome) and maturation (i.e., if there is a long time before and after the intervention, the participants have grown older and more mature), we used the shortest time gap possible between pre-test (1 week before the sessions) and the post-test (1 month after); we did not use a cognitive test, so the testing effect (i.e., the fact that the questions might be familiar and therefore easier after the interventions) is unlikely to appear; and we eliminated the instrumentation effect (i.e., using different people to score or rate the pre-experimental and post-experimental measurements) by having the questions scored by the same people (i.e., the girls) before and after the role-model interventions. Finally, regarding statistical regression toward the mean, although strictly speaking the sample was not selected randomly, we consider that because it comprised girls from different regions and socio-economic status in Spain from both public and private schools, it should include a variety of attitudes and opinions regarding the questions asked.

Once the possible effectiveness of the role-model intervention was analyzed through this design, an additional multigroup SEM analysis was carried out to test whether the counterstereotypical content of the sessions could act as a strength moderator of the changes found in the models’ relation(s) after the role-model sessions. A multigroup model nested in the post-test model was run using the girls’ perception of the role models’ reporting about the need for counterstereotypical skills (e.g., social and communication abilities) among the requirements for following a STEM career as a grouping variable.

### Measures

Data were collected using a reduced version of the expectancy–value questionnaire (EVQ). The EVQ is an empirically validated survey (Eccles and Wigfield, 1995) developed to measure career aspirations and educational choices. Following Eccles and Harold (1991) and Eccles and Wigfield (1995), all items were measured on a seven-point Likert scale (where 1 indicated “strongly disagree” and 7 “strongly agree”). The original items from the EVQ were translated into Spanish and two members of the research team made a back translation. Once this back translation was ready, to identify potential issues with the survey design that might lead to practical problems with implementation, a pilot study was carried out between April and June 2018. We recruited girls who were aged among 12–16, from six Spanish Schools in Cadiz, Malaga, and Madrid, which had previously attended the role models’ sessions to be sure the participants belonged to the same target group of the main study. The final sample for the first stage of the pilot was 126 students, but it decreased to 38 in the second wave.

The participants completed the questionnaires in the same way that it would be completed in the actual project (i.e., through an online platform). Once they had completed the two designed questionnaires, we found no significant problems on the survey design, except for the low rate of participation in the second wave. To address this problem in the main study, we asked for collaboration to the call center in charged with communication with the schools to track more closely the participation of the schools in both waves and to insist to the teachers of those that hadn’t answered yet, of the importance of transmitting to their students the need of answering the second questionnaire. As a result, in the main study the drop out ratio between waves was negligible.

#### Gender Stereotypes About Math Abilities

The gender stereotypes revolved around the higher math abilities and motivation of boys compared to girls (Li, 2007). Three items were used: “Math is more important for boys,” “Boys do better in math than girls,” and “In the future, math will be more useful for boys.”

#### Expectations of Math Success

The following seven items measuring several aspects related to girls’ expectations of success in math from expectancy–value theory were translated into Spanish (Eccles and Wigfield, 1995). Students had to rate their degree of agreement with the following statements: “I am talented at math”; “I expect to do well in a STEM degree”; “Math is easy for me”; “Learning new things in math is easy for me”; “I am more talented at math than other students in my class”; “I am more talented at math than at other subjects”; “I expect to do well at math this year.”

#### Math Enjoyment

Students had to rate their math enjoyment using the following five items, translated into Spanish: “I like math”; “I find math enjoyable”; “I learn a lot of interesting things in math”; “I like to solve math problems”; “I enjoy doing math exercises” (Wigfield and Eccles, 2002).

#### Importance Attached to Math

Students had to rate their level of agreement with seven items measuring the attainment and utility attached to math: “Mathematical skills increase job opportunities” (Wigfield and Eccles, 1992); “Mathematical skills will allow me to choose the work/career that I want”; “Mathematical skills are useful in the everyday world”; “Math is more useful than other subjects”; “I have always wanted to do well in math”; “I prefer doing well in math rather than in other subjects”; “Doing well at math makes me feel good” (Wigfield and Eccles, 2002).

#### STEM Career Choice

Students had to rate their likelihood of choosing a university degree across the following four STEM disciplines: math, physical science, computer science, and engineering (Stoeger et al., 2016). We created a construct that includes all the disciplines that usually configure the STEM field because we are interested in the overall result of all the degrees, not in a single specific one.

#### STEM Counterstereotypical Content of the Sessions: Highly Counterstereotypical Sessions Versus Lower Counterstereotypical Sessions

Because we could not manipulate the stereotypical content of the role models by using variable tuning, we included three items in the post-test questionnaire to measure the degree to which the girls perceived the role models as more counterstereotypical: “This profession requires communication skills,” “This profession requires teamwork,” and “This profession requires social skills.” We chose these three questions because they show skills (such as communication and teamwork) that are more congruent with the female gender role (social abilities).

Next, we grouped the after-session data into two subsets to test the counterstereotypical content of the sessions as a possible strength moderator of the relationships that change after the interventions. A confirmatory factor analysis (CFA) was conducted (Table 1) on the three questions. The factorial score was transformed into a dummy variable, using its median as a cut point. This produced a balanced split of the sample, with 50% of the cases having a value equal to 1 (Rigdon et al., 1998).

**TABLE 1:** Confirmatory factor analysis (CFA) of counter-stereotypical content of the sessions.

	Question	Loadings	AVE	Cronbach alpha
Counter-stereotypical content			0.679	0.858
Talk1	This profession requires teamwork	0.932		
Talk2	This profession requires social skills	0.788		
Talk3	This profession requires communication skills	0.740		

#### Construct Validity

Construct reliability assessment routinely focuses on composite reliability as an estimate of a construct’s internal consistency (Hair et al., 2011). Composite reliability values of 0.70–0.90 are regarded as satisfactory (Nunnally and Bernstein, 1994), whereas values below 0.60 indicate a lack of reliability. All the constructs in the present study have values over 0.8, well above the suggested threshold value. Likewise, each indicator’s absolute standardized loading should be higher than 0.70. Generally, indicators with loadings of 0.40–0.70 should be considered for removal from the scale only if doing so increases the composite reliability above the suggested threshold value (Hair et al., 2011). In the present study, all the items have loadings above or very near the cut-off value of 0.7. Only one item has a lower value (i.e., computing at 0.58), but deleting it does not increase the composite reliability of the construct STEM choice (0.825) (Hair et al., 2011).

The validity assessment of reflective measurement models focuses on convergent and discriminant validity. Researchers must examine the average variance extracted (AVE) for convergent validity. An AVE value of 0.50 or higher indicates a sufficient degree of convergent validity, meaning that the latent variable explains more than half of its indicators’ variance (Hair et al., 2011). As Table 2 shows, all the constructs have AVE values of at least 0.5 or very close to this cut-off (the lowest one corresponds to the construct “STEM choice”).

**TABLE 2:** CFA factor loadings, AVE, Cronbach’s alpha reliabilities, and cross loadings.

					Cronbach alpha	Cross correlations				
		Question	Loadings	AVE		Enjoyment	Importance	Stereotype	Expectations	STEM choice
Enjoyment				0.751	0.939					
	Enj1	I like math	0.865				0.509	−0.178	0.633	0.511
	Enj2	I like to solve math problems	0.867				0.534	−0.178	0.611	0.517
	Enj3	I learn a lot of interesting things in math	0.809				0.506	−0.183	0.527	0.489
	Enj4	I find math enjoyable	0.908				0.502	−0.176	0.597	0.502
	Enj5	I enjoy doing math exercises	0.881				0.521	−0.180	0.638	0.512
Importance				0.518	0.895					
	Ut1	Mathematical skills will allow me to choose the work/career that I want	0.765			0.499		−0.288	0.516	0.465
	Ut2	Mathematical skills are useful in everyday world	0.733			0.471		−0.245	0.437	0.375
	Ut3	Math is more useful than other subjects	0.725			0.386		−0.244	0.348	0.313
	Ut4	Mathematical skills increase job opportunities	0.791			0.430		−0.257	0.481	0.355
	Att1	Doing well in math makes me feel good	0.681			0.453		−0.274	0.398	0.364
	Att2	I have always wanted to do well in math	0.674			0.411		−0.278	0.327	0.354
	Att3	I prefer doing well in math than in other subjects	0.660			0.405		−0.226	0.369	0.286
Stereotype				0.682	0.865					
	St1	Boys do better in math than girls	0.860			−0.211	−0.298		−0.249	−0.320
	St2	In the future, math will be more useful for boys	0.848			−0.131	−0.298		−0.262	−0.290
	St3	Math is more important for boys	0.765			−0.153	−0.228		−0.222	−0.267
Expectations				0.751	0.956					
	Exp1	I am talented at math	0.905			0.646	0.536	−0.288		0.662
	Exp2	I expect to do well in a STEM degree	0.867			0.643	0.524	−0.245		0.617
	Exp3	Math is easy for me	0.888			0.675	0.510	−0.244		0.654
	Exp4	Learning new things in math is easy for me	0.889			0.657	0.535	−0.257		0.650
	Exp5	I am more talented at math than other students in my class	0.837			0.585	0.492	−0.274		0.608
	Exp6	I am more talented at math than in other subjects	0.839			0.630	0.455	−0.278		0.579
	Exp7	I expect to do well at math this year	0.839			0.549	0.532	−0.226		0.590
STEM_choice				0.481	0.824					
	Maths	I am considering math as a career for the future	0.774			0.512	0.424	−0.258	0.586	
	Physics	I am considering physics as a career for the future	0.789			0.424	0.400	−0.309	0.504	
	Engineering	I am considering engineering as a career for the future	0.605			0.334	0.354	−0.230	0.419	
	Computing	I am considering computing as a career for the future	0.581			0.253	0.252	−0.171	0.303	

The Fornell–Larcker criterion (Fornell and Larcker, 1981) was followed for the assessment of discriminant validity, where a latent construct shares more variance with its assigned indicators than with another latent variable in the structural model. The AVE of each latent construct should therefore be greater than the latent construct’s highest squared correlation with any other latent construct. Similarly, another more liberal criterion is met for every single item. Congruently, an indicator’s loading with its associated latent construct should be higher than its loadings with all the remaining constructs (i.e., the cross loadings).

## Results

All the analyses were conducted with the SEM in Stata 15.1. Several indicators of model fit were used, including χ^2^/df, the root mean square error of approximation (RMSEA), the Tucker–Lewis index (TLI), and the comparative fit index (CFI). General guidelines for the cut-off values of the different indicators suggest that an adequate fit is supported by RMSEA < 0.06, CFI > 0.90, TLI > 0.90, and χ^2^/df < 2 (e.g., Byrne, 1998; Hu and Bentler, 1999; Raykov and Marcoulides, 2000). All the models presented herein satisfy these conditions and were estimated with full information maximum likelihood to incorporate cases with missing data (Enders, 2010). Robust standard errors were clustered by schools. We provide correlation matrices for replicability purposes in Tables 3A,B. Table 3A shows the correlation matrix for the all the variables before and after the role model sessions, whereas, Table 3B displays the correlations for the same variables but for low and high counterstrerotypical sessions within the post-intervention sample.

**TABLE 3A:** Correlation matrix.

Variables	1	2	3	4	5	6	7	8	9	10	11	12	13	14	15	16	17	18	19	20	21	22	23	24	25	26	Mean (*t* = 1)	*SD* (*t* = 1)
Enj1	1	0.777	0.679	0.800	0.768	0.423	0.393	0.336	0.369	0.387	0.342	0.363	-0.157	-0.112	-0.121	0.538	0.558	0.595	0.536	0.529	0.545	0.458	0.449	0.337	0.300	0.179	4.37	1.78
Enj2	0.740	1	0.627	0.746	0.760	0.459	0.431	0.353	0.397	0.417	0.393	0.432	-0.187	-0.134	-0.190	0.468	0.522	0.538	0.498	0.512	0.509	0.409	0.439	0.357	0.328	0.213	4.09	1.87
Enj3	0.722	0.691	1	0.751	0.674	0.386	0.439	0.341	0.396	0.418	0.411	0.260	-0.199	-0.113	-0.111	0.429	0.435	0.442	0.457	0.378	0.337	0.362	0.356	0.328	0.241	0.242	4.86	1.69
Enj4	0.781	0.778	0.768	1	0.798	0.414	0.390	0.361	0.355	0.418	0.366	0.370	-0.149	-0.062	-0.185	0.492	0.529	0.538	0.511	0.464	0.508	0.380	0.405	0.318	0.263	0.184	4.50	1.71
Enj5	0.746	0.771	0.689	0.812	1	0.437	0.419	0.352	0.408	0.430	0.400	0.371	-0.158	-0.114	-0.188	0.501	0.516	0.539	0.543	0.497	0.523	0.400	0.393	0.332	0.282	0.185	4.37	1.86
Ut1	0.359	0.349	0.387	0.362	0.376	1	0.552	0.577	0.687	0.490	0.507	0.514	-0.216	-0.242	-0.161	0.430	0.476	0.394	0.425	0.400	0.352	0.358	0.363	0.309	0.374	0.223	4.95	1.91
Ut2	0.381	0.385	0.458	0.376	0.387	0.553	1	0.556	0.643	0.538	0.569	0.390	-0.270	-0.191	-0.167	0.330	0.326	0.305	0.357	0.329	0.249	0.345	0.272	0.234	0.240	0.131	5.36	1.61
Ut3	0.262	0.261	0.254	0.288	0.306	0.513	0.516	1	0.680	0.538	0.549	0.539	-0.171	-0.226	-0.127	0.308	0.340	0.260	0.316	0.290	0.325	0.285	0.233	0.271	0.212	0.146	4.94	1.76
Ut4	0.343	0.329	0.365	0.325	0.321	0.626	0.603	0.587	1	0.559	0.591	0.494	-0.224	-0.298	-0.179	0.429	0.447	0.378	0.391	0.369	0.338	0.376	0.268	0.255	0.233	0.131	5.09	1.70
At1	0.314	0.284	0.347	0.324	0.322	0.551	0.526	0.443	0.499	1	0.608	0.419	-0.206	-0.173	-0.192	0.266	0.311	0.295	0.273	0.265	0.265	0.311	0.248	0.232	0.219	0.158	5.08	1.70
At2	0.314	0.304	0.353	0.337	0.359	0.461	0.596	0.500	0.479	0.546	1	0.412	-0.231	-0.158	-0.127	0.264	0.282	0.258	0.318	0.221	0.191	0.281	0.292	0.232	0.226	0.232	5.44	1.67
At3	0.312	0.297	0.312	0.356	0.324	0.526	0.463	0.560	0.480	0.452	0.439	1	-0.156	-0.200	-0.116	0.256	0.292	0.286	0.293	0.293	0.311	0.143	0.213	0.172	0.213	0.084	4.76	1.89
St1	-0.158	-0.149	-0.223	-0.178	-0.152	-0.197	-0.206	-0.155	-0.188	-0.207	-0.249	-0.103	1	0.713	0.612	-0.177	-0.165	-0.133	-0.196	-0.123	-0.070	-0.161	-0.199	-0.279	-0.226	-0.127	1.31	0.90
St2	-0.116	-0.110	-0.133	-0.110	-0.112	-0.193	-0.172	-0.161	-0.206	-0.176	-0.156	-0.080	0.722	1	0.627	-0.232	-0.217	-0.169	-0.221	-0.160	-0.133	-0.206	-0.197	-0.185	-0.202	-0.102	1.37	1.04
St3	-0.084	-0.078	-0.072	-0.105	-0.088	-0.133	-0.186	-0.102	-0.133	-0.152	-0.169	-0.073	0.680	0.645	1	-0.197	-0.226	-0.183	-0.247	-0.147	-0.167	-0.189	-0.180	-0.191	-0.113	-0.104	1.42	0.99
Exp1	0.642	0.598	0.590	0.641	0.640	0.509	0.404	0.293	0.396	0.373	0.297	0.408	-0.191	-0.221	-0.125	1	0.809	0.808	0.811	0.805	0.782	0.800	0.548	0.468	0.440	0.273	4.14	1.87
Exp2	0.603	0.611	0.580	0.602	0.616	0.481	0.324	0.268	0.389	0.365	0.268	0.340	-0.207	-0.172	-0.105	0.779	1	0.767	0.770	0.756	0.789	0.718	0.526	0.426	0.426	0.237	3.92	1.93
Exp3	0.650	0.610	0.603	0.620	0.597	0.420	0.328	0.213	0.374	0.338	0.250	0.387	-0.184	-0.199	-0.118	0.802	0.763	1	0.827	0.777	0.798	0.710	0.575	0.491	0.450	0.298	3.72	1.81
Exp4	0.650	0.627	0.595	0.598	0.654	0.491	0.427	0.326	0.419	0.383	0.314	0.424	-0.210	-0.209	-0.163	0.793	0.772	0.809	1	0.766	0.733	0.731	0.563	0.472	0.431	0.329	4.04	1.88
Exp5	0.561	0.535	0.453	0.476	0.528	0.367	0.327	0.228	0.342	0.300	0.198	0.308	-0.159	-0.171	-0.087	0.760	0.722	0.751	0.754	1	0.775	0.708	0.529	0.467	0.471	0.260	3.54	1.93
Exp6	0.605	0.578	0.474	0.593	0.598	0.366	0.280	0.261	0.297	0.331	0.187	0.397	-0.133	-0.141	-0.117	0.758	0.736	0.742	0.712	0.733	1	0.675	0.530	0.417	0.433	0.190	3.61	2.01
Exp7	0.544	0.521	0.533	0.553	0.538	0.452	0.442	0.340	0.433	0.422	0.349	0.377	-0.245	-0.237	-0.216	0.769	0.704	0.700	0.708	0.649	0.637	1	0.463	0.393	0.351	0.268	4.38	1.81
Maths	0.493	0.500	0.497	0.509	0.512	0.410	0.341	0.224	0.284	0.357	0.271	0.309	-0.238	-0.232	-0.212	0.538	0.568	0.520	0.532	0.478	0.510	0.477	1	0.585	0.566	0.493	3.37	1.92
Physics	0.383	0.400	0.433	0.409	0.395	0.356	0.290	0.219	0.236	0.322	0.268	0.198	-0.293	-0.279	-0.271	0.486	0.435	0.440	0.444	0.407	0.386	0.452	0.616	1	0.589	0.578	3.52	2.03
Engin	0.244	0.250	0.316	0.334	0.306	0.314	0.308	0.123	0.161	0.209	0.281	0.158	-0.216	-0.184	-0.173	0.409	0.296	0.282	0.286	0.273	0.206	0.385	0.374	0.490	1	0.499	3.82	2.12
Comput	0.223	0.173	0.322	0.222	0.233	0.231	0.199	0.169	0.196	0.222	0.245	0.073	-0.178	-0.101	-0.161	0.285	0.231	0.288	0.277	0.191	0.101	0.282	0.433	0.525	0.295	1	3.09	2.05
Mean (*t* = 0)	3.58	3.30	3.96	3.66	3.72	4.20	4.72	4.29	4.40	4.30	4.75	3.73	1.76	1.80	1.95	3.78	3.32	3.32	3.53	3.01	3.09	4.07	2.28	2.50	2.50	2.45		
*SD* (*t* = 0)	1.73	1.64	1.68	1.67	1.75	1.84	1.71	1.69	1.62	1.73	1.74	1.71	1.36	1.46	1.50	1.84	1.84	1.74	1.76	1.87	1.84	1.73	1.61	1.81	1.85	1.78		

*t = 0 (bottom-left triangle) and t = 1 (top-right triangle).*

**TABLE 3B:** Correlation matrix.

Variables	1	2	3	4	5	6	7	8	9	10	11	12	13	14	15	16	17	18	19	20	21	22	23	24	25	26	Mean (high)	*SD* (high)
Enj1	1	0.781	0.726	0.851	0.794	0.490	0.436	0.377	0.381	0.469	0.450	0.406	-0.035	0.055	0.053	0.620	0.656	0.669	0.579	0.626	0.630	0.491	0.519	0.395	0.443	0.229	4.61	1.85
Enj2	0.770	1	0.725	0.787	0.729	0.476	0.482	0.444	0.457	0.505	0.512	0.437	-0.105	-0.030	-0.063	0.557	0.628	0.630	0.543	0.568	0.610	0.481	0.528	0.446	0.427	0.251	4.32	1.95
Enj3	0.628	0.501	1	0.823	0.762	0.408	0.539	0.369	0.394	0.470	0.482	0.342	-0.027	0.106	0.027	0.493	0.542	0.559	0.514	0.504	0.450	0.394	0.394	0.363	0.315	0.314	4.96	1.77
Enj4	0.706	0.688	0.640	1	0.862	0.430	0.465	0.419	0.358	0.502	0.462	0.372	-0.042	0.113	-0.003	0.537	0.601	0.618	0.553	0.545	0.592	0.396	0.431	0.417	0.371	0.220	4.63	1.89
Enj5	0.703	0.789	0.551	0.680	1	0.441	0.438	0.410	0.414	0.514	0.478	0.372	0.003	0.049	-0.004	0.554	0.599	0.598	0.573	0.566	0.595	0.441	0.423	0.388	0.323	0.179	4.61	1.92
Ut1	0.323	0.434	0.292	0.395	0.421	1	0.571	0.561	0.679	0.467	0.465	0.509	-0.105	-0.253	-0.084	0.498	0.522	0.475	0.503	0.484	0.437	0.403	0.428	0.403	0.371	0.213	5.03	1.99
Ut2	0.351	0.339	0.289	0.276	0.386	0.522	1	0.588	0.620	0.498	0.584	0.476	-0.082	0.005	0.076	0.347	0.349	0.361	0.415	0.350	0.295	0.354	0.305	0.323	0.260	0.164	5.46	1.63
Ut3	0.258	0.177	0.270	0.246	0.230	0.611	0.503	1	0.699	0.471	0.521	0.553	-0.096	-0.215	-0.065	0.363	0.423	0.319	0.410	0.354	0.403	0.354	0.286	0.318	0.232	0.120	4.99	1.75
Ut4	0.307	0.287	0.360	0.330	0.393	0.696	0.695	0.662	1	0.536	0.567	0.559	-0.080	-0.270	-0.071	0.491	0.506	0.466	0.484	0.464	0.428	0.454	0.328	0.371	0.231	0.200	5.15	1.75
At1	0.284	0.269	0.337	0.294	0.308	0.502	0.540	0.607	0.603	1	0.621	0.419	-0.025	-0.082	-0.049	0.348	0.365	0.439	0.342	0.340	0.371	0.367	0.330	0.319	0.218	0.212	5.27	1.67
At2	0.232	0.260	0.285	0.246	0.302	0.551	0.506	0.586	0.673	0.616	1	0.519	-0.170	-0.060	0.073	0.324	0.387	0.359	0.386	0.299	0.296	0.323	0.356	0.319	0.239	0.239	5.58	1.70
At3	0.305	0.374	0.137	0.361	0.357	0.577	0.248	0.533	0.419	0.438	0.334	1	-0.156	-0.239	-0.139	0.351	0.335	0.329	0.384	0.357	0.335	0.248	0.321	0.314	0.324	0.163	4.60	1.87
St1	-0.258	-0.259	-0.274	-0.213	-0.262	-0.262	-0.336	-0.197	-0.297	-0.263	-0.126	-0.168	1	0.524	0.541	-0.081	-0.095	-0.121	-0.152	-0.139	-0.147	-0.132	-0.110	-0.217	-0.119	0.004	1.17	0.62
St2	-0.264	-0.223	-0.250	-0.209	-0.231	-0.162	-0.242	-0.200	-0.268	-0.146	-0.062	-0.160	0.708	1	0.674	-0.221	-0.177	-0.167	-0.220	-0.201	-0.212	-0.220	-0.125	-0.158	-0.093	-0.028	1.25	0.86
St3	-0.253	-0.270	-0.229	-0.358	-0.308	-0.211	-0.314	-0.121	-0.280	-0.320	-0.210	-0.127	0.576	0.539	1	-0.148	-0.180	-0.162	-0.199	-0.154	-0.232	-0.125	-0.063	-0.158	-0.062	0.019	1.25	0.76
Exp1	0.381	0.334	0.316	0.405	0.396	0.373	0.323	0.250	0.346	0.148	0.217	0.196	-0.225	-0.235	-0.280	1	0.815	0.820	0.824	0.811	0.789	0.798	0.584	0.468	0.475	0.246	4.45	1.85
Exp2	0.374	0.357	0.261	0.400	0.376	0.447	0.304	0.285	0.366	0.234	0.191	0.300	-0.249	-0.291	-0.375	0.791	1	0.794	0.794	0.770	0.794	0.713	0.598	0.509	0.458	0.270	4.17	2.05
Exp3	0.460	0.398	0.252	0.398	0.443	0.283	0.226	0.228	0.230	0.138	0.156	0.276	-0.123	-0.153	-0.225	0.802	0.720	1	0.830	0.781	0.792	0.696	0.609	0.565	0.531	0.347	3.95	1.87
Exp4	0.422	0.421	0.333	0.425	0.473	0.324	0.314	0.245	0.241	0.230	0.257	0.239	-0.219	-0.213	-0.341	0.816	0.750	0.818	1	0.789	0.728	0.693	0.612	0.555	0.500	0.348	4.33	1.96
Exp5	0.373	0.404	0.242	0.349	0.388	0.346	0.325	0.246	0.253	0.157	0.194	0.226	-0.169	-0.182	-0.241	0.811	0.724	0.808	0.786	1	0.737	0.693	0.584	0.535	0.560	0.277	3.78	2.03
Exp6	0.383	0.329	0.206	0.371	0.403	0.294	0.241	0.275	0.230	0.156	0.141	0.305	-0.141	-0.189	-0.251	0.815	0.805	0.854	0.799	0.845	1	0.642	0.591	0.475	0.513	0.218	3.80	2.15
Exp7	0.430	0.378	0.341	0.401	0.395	0.350	0.387	0.286	0.312	0.259	0.266	0.174	-0.149	-0.188	-0.263	0.828	0.727	0.760	0.818	0.788	0.801	1	0.494	0.376	0.383	0.251	4.81	1.72
Maths	0.277	0.204	0.246	0.315	0.205	0.257	0.182	0.173	0.221	0.112	0.105	0.054	-0.206	-0.294	-0.235	0.481	0.419	0.500	0.456	0.454	0.472	0.481	1	0.694	0.678	0.565	3.81	2.12
Physics	0.202	0.184	0.231	0.128	0.195	0.187	0.092	0.190	0.105	0.052	0.072	0.038	-0.403	-0.251	-0.289	0.419	0.269	0.397	0.358	0.345	0.314	0.394	0.344	1	0.671	0.603	3.90	2.11
Engin	0.047	0.166	0.050	0.063	0.174	0.361	0.120	0.161	0.202	0.086	0.124	0.027	-0.290	-0.269	-0.190	0.429	0.417	0.352	0.336	0.407	0.381	0.385	0.409	0.487	1	0.513	3.98	2.22
Comput	0.163	0.230	0.187	0.197	0.237	0.291	0.116	0.265	0.120	0.098	0.205	0.117	-0.292	-0.273	-0.280	0.380	0.264	0.300	0.355	0.307	0.268	0.359	0.374	0.628	0.510	1	3.33	2.17
Mean (low)	4.17	4.02	4.78	4.43	4.23	4.88	5.30	4.97	5.07	4.98	5.27	5.15	1.42	1.46	1.55	3.79	3.65	3.41	3.71	3.32	3.42	3.84	2.84	3.02	3.62	2.75		
*SD* (low)	1.66	1.71	1.56	1.47	1.71	1.82	1.55	1.74	1.64	1.58	1.56	1.79	0.93	1.06	1.02	1.88	1.77	1.76	1.73	1.78	1.82	1.81	1.47	1.83	1.93	1.80		

*Low Counterstereotypical (bottom-left triangle) and High Counterstereotypical (top-right triangle) for t = 1.*

### Measurement Models and Invariance

**FIGURE 3:**
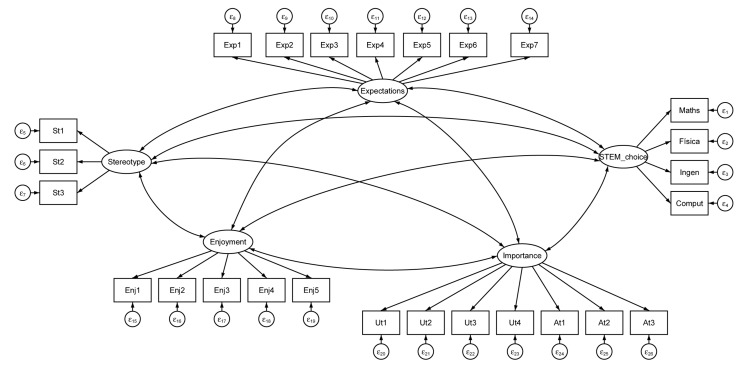
Confirmatory factor analysis (CFA) with latent variables.

To assess the invariances between the two points in time (before and after the role-model sessions), a measurement model was estimated, including the five constructs in Figure 3, because they are focal constructs in the following STEM-choice models. The five constructs included in the measurement model were gender stereotypes, expectations of success, enjoyment, importance, and STEM choice. All constructs were specified as latent variables, and the covariances between all five constructs were estimated.

The unconstrained multigroup CFAs (model 1) showed adequate model fits across a range of frequently emphasized fit statistics for the latent constructs (χ^2^/df = 1.936; RMSEA = 0.055; TLI = 0.947; CFI = 0.953).

After confirming the goodness of fit of the CFAs, the invariances between the two moments were explored. The sequence of analyses began with a combined multiple-group model with no cross-time equality constraints for the five latent constructs before and after the role-model sessions (model 1). Second, the constraint that item loadings are invariant between the two moments was added (model 2). Third, the constraint that loadings as well as item error variances are equivalent across samples was added (model 3). Finally, the constraint that loadings as well as intercepts are equivalent across samples was added (model 4). The nested models were compared according to the change in the χ^2^ statistic relative to that in the degrees of freedom; a significant worsening of model fit indicates that the imposed model constraints are not tenable.

The model fits for sequential constrained models 1–4 for each of the latent constructs are given in Table 4. The fit statistics of models 1 and 2 (the unconstrained and loading-invariant models, respectively) are acceptable, and the change in χ^2^ is not statistically significant. This result implies that the condition of partial scalar invariance is therefore met (e.g., Byrne, 2010), indicating that the time difference does not differentially affect the underlying measurement characteristics of the constructs; i.e., the constructs have the same meaning before and after attending the role-model sessions, and quantitative comparisons of factor scores can be undertaken meaningfully at both points in time. The factor loadings, which are all statistically significant, are presented in Table 2.

**TABLE 4:** Fit statistics for sequential constrained models.

Model	Chi squared	df	Chi squared/df	Comparison	Chi squared	df	*p*-value	RMSEA	CFI	TLI
(1) Same form model	1,118.8	578	1.936					0.055	0.953	0.947
**(2) Equal loadings model**	**1,143.7**	**599**	**1.909**	**1 vs. 2**	**24.84**	**21**	**0.254**	**0.055**	**0.953**	**0.949**
(3) Equal loadings and error variances model	1,250.4	625	2.001	2 vs. 3	106.72	26	0.000	0.057	0.946	0.944
(4) Equal loadings and cons model	1,285.1	625	2.056	2 vs. 4	141.42	26	0.000	0.059	0.943	0.940

*Selected model in bold.*

Model 3 (loading and error-variance invariant) and model 4 (loading and intercept invariant) cannot be accepted because of a statistically significant worsening in the change in χ^2^ with respect to model 2. This implies that the heterogeneity and mean values of the constructs changed after the role-model sessions, indicating (as will be shown later) the effectiveness of these interventions in changing the motivational factors, gender stereotypes, and STEM choice of girls.

### Testing the Theoretical STEM-Choice Model (H1)

**FIGURE 4:**
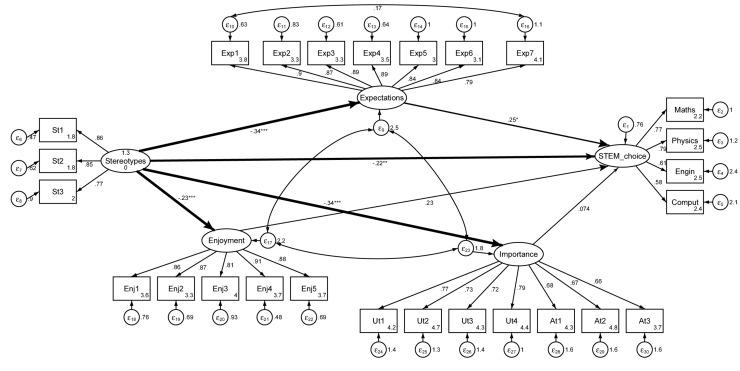
STEM-choice model before the role-model sessions.

Having ensured the partial scalar invariance and the consistency of the constructs before and after the role-model sessions, path models were used to test the theoretical STEM-choice model. The model includes all of the paths and covariances shown in Figure 4, as well as paths estimating the predictive relations between gender stereotypes and the motivational constructs (i.e., expectations of success, enjoyment, importance, and STEM choice) shown in Figure 3.

**FIGURE 5:**
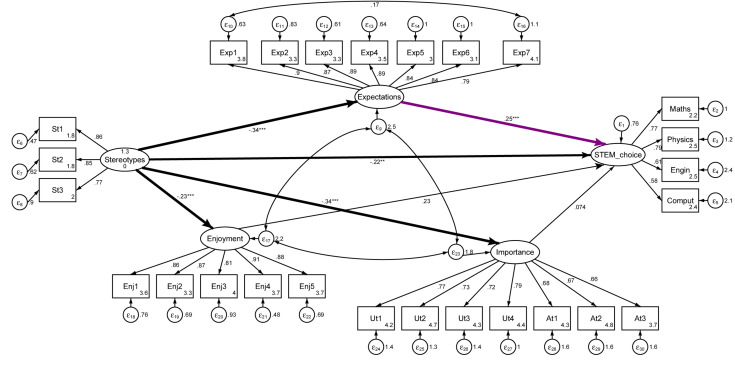
SEM-choice model after the role-model sessions.

The STEM-choice path model fits the data well (see Table 5). This discussion concentrates on the relationships depicted in Figure 4 (i.e., proposed in the theoretical model) because they are the focus of H1. Overall, the findings confirm, at least partially, the hypothesized relationships for the STEM-choice model (Figure 5). Stereotypes have a negative significant (direct) effect at 5% on STEM choices, although it will be seen later that the total effect is stronger and highly significant. Expectations of success have the highest and most-significant positive effect on STEM choice (although only marginal before the role-model interventions). However, there is no evidence supporting the positive influence of enjoyment and importance on STEM choice, these being because these constructs seem to have no significant effect on girls’ interests in choosing a STEM career.

**TABLE 5:** Estimated path coefficients (final model).

	*t* = 0				*t* = 1					
Path coefficient	*b*	*B*	St. Dv.		*b*	*B*	St. Dv.		LR test	
Stereotype → enjoyment	−0.179	−0.232	(0.051)	***	−0.464	−0.406	(0.136)	***	1.25	
Stereotype → importance	−0.274	−0.337	(0.093)	***	−0.336	−0.633	(0.121)	***	4.27	**
Stereotype → expectations	−0.248	−0.341	(0.063)	***	0.233	−0.537	(0.207)	***	1.52	
Stereotype → STEM choice	−0.205	−0.218	(0.084)	**	−0.128	−0.253	(0.149)	**	0.05	
Enjoyment → STEM choice	0.277	0.227	(0.185)		0.109	0.106	(0.134)		1.05	
Importance → STEM choice	0.086	0.074	(0.072)		0.023	0.024	(0.121)		0.21	
Expectations → STEM choice	0.327	0.253	(0.114)	*	0.557	0.478	(0.095)	***	4.54	**
Cov (enjoyment, importance)	0.544	1.102	(0.151)	***						
Cov (enjoyment, expectations)	0.767	1.743	(0.231)	***						
Cov (expectations, importance)	0.530	1.116	(0.162)	***						
Chi2 (601)	1,120									
Chi(2)/df	1.863									
RMSEA	0.053									
CFI	0.955									
TLI	0.951									
R2 overall	0.870									

Invariant loadings and covariance between interest and enjoyment, mean(stereotypes) = 0. Robust standard errors clustered by school. b = standardized path coefficient. B = unstandardized path coefficient. *, **, *** represent 10%, 5%, 1% significance levels respectively.

#### Total and Indirect Effects of Role Stereotypes

In the STEM-choice model, stereotypes about math ability have both a direct and indirect influence on STEM choice, and so direct and indirect effects were tested through the motivational constructs of enjoyment, importance, and expectations of success with indirect effects in Stata. Table 6 shows the total effect, together with one direct and three indirect effects that make up the total effect.

**TABLE 6:** Total, direct, and indirect effects for stereotypes.

	*t* = 0			*t* = 1		
Path coefficient	*b*	St. Dv.		*b*	St. Dv.	
Stereotype → STEM choice						
Total effect	−0.381	(0.084)	***	−0.568	(0.207)	***
Total indirect effect	−0.164	(0.045)	***	−0.315	(0.081)	***
Via Enjoyment	−0.049			−0.057		
Via Importance	−0.016			−0.006		
Via Expectations	−0.055			−0.121		
Direct effect	−0.218	(0.084)	**	−0.253	(0.149)	**

Invariant loadings and covariance between interest and enjoyment, mean(stereotypes) = 0. b = standardized path coefficient. Robust standard errors clustered by school. *, **, *** represent 10%, 5%, 1% significance levels respectively.

The results suggest the indirect effects of stereotypes about math abilities on STEM choice via the three motivational factors of the model. As seen in Table 6, the total effect of stereotypes about math abilities on the STEM-choice model is negative and highly significant. This is due to adding the indirect effect of stereotypes via enjoyment, importance, and especially expectations of success to the direct effect of the construct. The indirect effects thus suggest that although stereotypes about math abilities have a significant direct effect on STEM choice at 5%, their total effect (especially via expectations) is highly significant both before and after the role-model interventions.

### Testing Mean-Level Differences in Research Variables (H2a)

First, differences in student motivations and gender stereotypes about math abilities after the role-model sessions were examined by using univariate *t*-test scores (Table 7). As anticipated in the CFA model, there are several significant differences across time. The variables of enjoyment, importance, expectations of success, and STEM choice increase significantly after attending the role-model sessions. Conversely, stereotypes regarding women’s lower math abilities decrease significantly after attending the role-model sessions. These findings are consistent with the predictions made in H2a.

**TABLE 7:** Means and univariate *t*-test scores before and after the role-model sessions.

Latent variable	Difference	Std. err	*t* stat	*p*-value
Enjoyment	0.769	0.121	6.336	0.000
Importance	0.766	0.111	6.935	0.000
Stereotypes	−0.419	0.075	−5.594	0.000
Expectations	0.503	0.134	3.768	0.000
STEM choice	0.791	0.103	7.691	0.000

*Estimated from model 3.*

### Testing the Moderator Effect of the Role-Model Sessions (H2b)

After changes in the mean value of the constructs were confirmed, whether the relations in the STEM-choice model (i.e., the path model described under H1) vary after the role-model sessions was also tested. Moderation of the role-model sessions through multigroup SEMs was also tested (Little, 2013). For these purposes, the change in χ^2^ (Δχ^2^) was examined across two nested models: one that freely estimated the predictive paths and covariances for each group separately, and another that constrained all or some of the predictive paths and covariances to be equal across two moments in time.

Differences in the path coefficients were tested using likelihood ratio tests (Table 5, LR test column). At both points in time, the estimated path coefficients kept their sign and similar significance. The outcomes suggested that there is an increase in the path coefficient from expectations to STEM choice that goes from marginally significant to highly significant after the role-model interventions. This result shows that the positive influence of expectations on STEM choice is reinforced after attending the role-model sessions, thus confirming H2b. A strengthening in the negative influence of stereotypes about math abilities on importance after the role-model sessions is also observed, which is also consistent with the moderator effect of the sessions predicted in H2b. This highlights the relevance of these interventions. That is, reducing the weight of stereotypes about math abilities has a strong effect on the importance that girls attribute to doing well in math, a basic competence in high demand in STEM careers.

### Testing the Counterstereotypical Content of the Sessions as a Moderator of Strength (H3)

To delve into the possible causes of the moderating effect of the role-model sessions, we examined the possible influence of a role model mentioning during a session that counterstereotypical skills are among the requirements for following a STEM career. Multigroup SEMs were run within the sample after the role-model sessions to evaluate the possible role of the counterstereotypical content of the sessions as a moderator of strength (Bentler, 1995) on the effect of expectations on STEM choices.

A dummy variable was used to group the sessions into two clusters, one comprising the sessions that the participants considered to be highly counterstereotypical regarding the demand for social and communication skills among STEM career requirements, and another comprising the remaining sessions that the participants perceived to be more stereotypical.

According to the results in Table 8, expectations and STEM choice increase significantly among girls who believe that the role-model sessions are highly counterstereotypical about STEM career requirements. Meanwhile, the stereotype construct regarding math abilities decrease significantly. Finally, there is no significant effect on importance and only a marginal effect on enjoyment.

**TABLE 8:** Means and univariate *t*-test scores between girls who perceived the role-model sessions as counter-stereotypical and those who did not.

Latent variable	Low counter-stereotypical	High counter-stereotypical	Difference	Std. err	*t* stat	*p*-value
Enjoyment	0.283	0.576	0.293	0.175	1.679	0.094
Importance	0.288	0.549	0.261	0.160	1.633	0.104
Stereotypes	−0.039	−0.273	−0.234	0.081	−2.876	0.004
Expectations	−0.044	0.539	0.582	0.192	3.030	0.003
STEM choice	0.158	0.718	0.560	0.158	3.538	0.001

*Estimated from model 3.*

After confirming the changes in the mean values of the constructs, the analysis concentrated on testing whether the relationships in the STEM-choice model after the role-model sessions vary between those girls who considered the role-model sessions to be highly counterstereotypical and those who considered the sessions to be more stereotypical. In particular, we tested whether there are significant changes in the path coefficient that measures the influence of expectations of success on STEM choice between the two groups of girls. As the results in Table 9 show, there is a significant increase in the path coefficient from expectations to STEM choice. Thus, we conclude that participant feedback on whether the sessions about STEM career requirements are counterstereotypical acts as a moderator, thus confirming H3.

**TABLE 9:** Estimated path coefficients with high/low counter-stereotypical groups for the post role-model sessions period.

	Low counter-stereotypical	High counter-stereotypical		
Path coefficient	*B*	*B*	LR test	
Stereotype → enjoyment	−0.491	−0.063	1.68	
Stereotype → importance	−0.652	−0.526	0.19	
Stereotype → expectations	−0.351	−0.783	1.67	
Stereotype → STEM choice	−0.468	−0.101	1.14	
Enjoyment → STEM choice	−0.055	0.186	2.04	
Importance → STEM choice	0.057	−0.069	0.57	
Expectations → STEM choice	0.334	0.631	4.29	**

*, **, *** represent 10%, 5%, 1% significance levels respectively.

**FIGURE 6:**
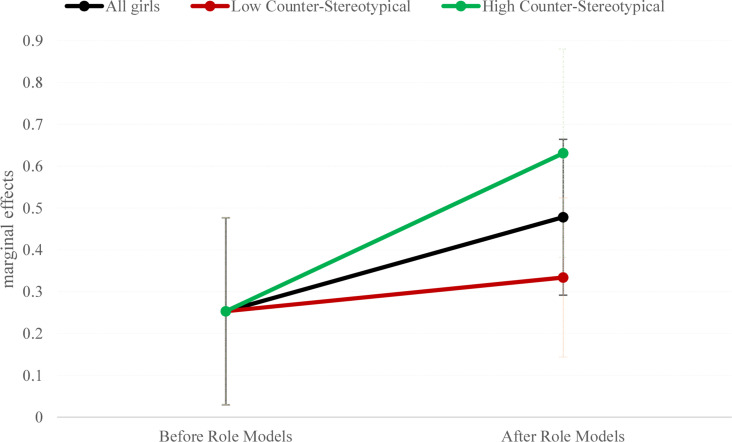
Marginal effects of expectations on STEM choice.

Finally, the marginal effect of expectations of success on STEM choice in both groups is shown in Figure 6. The effect of expectations on STEM choice after the intervention is between the minimum value for this path coefficient estimated from those girls who perceive the interventions as being more stereotypical and the maximum value obtained for those girls who consider the sessions to be highly counterstereotypical.

## Conclusion and Discussion

This research contributes to the literature on how to increase girls’ interest in STEM through a female role-model-based intervention. This study advances our understanding of the influence of female role models in improving girls’ preferences for STEM by exploring the change in the mean values of the constructs (i.e., mean-level group differences) and in their relationships (i.e., moderation) by using an adaptation of the expectancy–value model of career choice in STEM fields. The findings of this research show that the optimal way to encourage young girls to pursue emerging high-growth roles, particularly those requiring STEM math skills, is to expose them to the professional and personal experiences of actual female role models with a successful professional trajectory in STEM fields.

On average, the role-model sessions significantly increased the two considered task-value factors of the expectancy–value theory (i.e., enjoyment and importance), as well as girls’ expectations of success in math, together with girls’ preference for a STEM career. These sessions also contributed to decreasing the effect of gender-role stereotypes. Additionally, the female role-model sessions had a moderator effect in increasing the influence of expectations of success on STEM choices. In particular, when young girls perceive that counterstereotypical skills (such as teamworking, communication, and social skills) are among the requirements demanded across the different STEM professions, the positive effect that the expectation of success has on the intention to pursue a STEM career is reinforced. Thus, the counterstereotypical content of the sessions acted as a moderator because it strengthened the influence that expectations of success had on STEM choices.

This result could be because the impact of ability beliefs on STEM choice depends on the extent to which the stereotypes (resp. counterstereotypes) are incongruent (resp. congruent) with individuals’ self-concepts and goals (Starr, 2019). Indeed, according to the theory of role congruity (Diekman et al., 2010), social skills are more congruent with the communal goals (e.g., working with or helping other people) that women are more likely to endorse. Thus, in this case the concordance occurs when girls perceive that among the requirements for following a STEM career, which are usually thought mainly to include masculine agentic goals such as developing instrumental and technical tasks, there are counterstereotypical skills in this field (such as communication and social abilities). This congruence acts as a strength moderator of the positive impact of expectancy beliefs on STEM choice.

Of course, many other dimensions of the role-model sessions could also play a moderator role, but the sessions were designed especially to offer the girls firsthand information about the actual skills and abilities formally or informally needed to pursue a STEM career from the direct experience of a female expert in those fields. A wide range of studies have shown that the preferences for certain jobs and skill sets among men and women are shaped by both the expectation and experience of diversity and inclusion across occupations (Seron et al., 2016; Cardador, 2017; Kang et al., 2019). This could be due, in part, to the increase in the feelings of belonging and inclusion in these domains that they experience after having been exposed to female role models who are successful in STEM fields (Walton and Cohen, 2007; Shin et al., 2016; Casad et al., 2018; Kang et al., 2019; Van Camp et al., 2019).

These interventions also strengthen the link between stereotypes about math abilities and the importance that girls attach to a task highly related to STEM, such as doing math (Wigfield and Eccles, 2002). This suggests the relevance of these interventions because exposing girls to female role models who contradict stereotypical portrayals of people in STEM fields produces a greater increase in the subjective value (in terms of importance) that the girls participating in the intervention session attach to STEM subjects (Cheryan et al., 2015; Sáinz et al., 2019).

The analysis of the total and indirect effects of role stereotypes shows that congruent with expectations, stereotypes about math abilities have a negative total effect on girls’ intentions to choose a STEM field (Sáinz and Eccles, 2012). This effect is highly significant and stronger than the direct effect, especially via expectations of success. This latter result is explained by the negative drag that gender stereotypes have on girls’ expectations regarding their abilities and skills in a usually male-dominated world, such as that of many STEM fields (Rosenthal et al., 2011; Good et al., 2012; Shin et al., 2016). All of these authors agree that these stereotypes should be overcome because they could undermine the recruitment and retention of female STEM students who do not match these stereotypes.

The findings of the present study make several important contributions to the existing literature on role models and girls in STEM, which can help future research and policies on this topic. Much of the previous research was focused on undergraduate or high-school students (e.g., Anderson and Gilbride, 2003; Rosenthal et al., 2011; Shin et al., 2016; Van Camp et al., 2019), but the present research addresses girls from and above 12 years old because this is the age when their self-perception of competence and self-confidence begins to fall (Willms and Jacobsen, 1990; Sáinz and Eccles, 2012). This implies that future research should be focused on the start of the leaky pipeline, before students specialize and choose their different academic tracks in secondary education and beyond, which is especially relevant if the intention is to fix this problem from the very beginning. Another important contribution is the identification, through a one-group pretest–post-test design, of female role-model interventions as a way of reducing stereotypes and of boosting the motivational factors that play an important role in girls’ engagement with STEM fields. This type of design to evaluate the effectiveness of these role-model interventions is especially versatile, and although, in general, it still has some limitations, certain rules have been applied to mitigate the negative effects that could stem from the absence of a control group. The fact that girls find STEM careers more interesting after the role-model sessions is also worth mentioning. This is aligned with the literature on role-model interventions (Shin et al., 2016; Van Camp et al., 2019).

Finally, it is also important to highlight that the present role-model exposure was not carried out in an experimental or artificial environment (with avatars or online biographies) but rather is the consequence of an actual and innovative female role-model intervention implemented by a foundation with continuity over time and international expansion. Indeed, this program is currently being spread to many countries around the world (the United Kingdom, Spain, Serbia, Switzerland, Singapore, Italy, Mexico, Costa Rica, Chile, Peru, Brazil, Honduras, and Panama). These role-model sessions are carried out with actual successful women volunteers that are experts in their fields and are willing to collaborate with the program, and we consider that this creates an atmosphere of closeness and warmth that is ideal for the girls to interact directly with the role models and dare to share their doubts and concerns regarding the male-dominated domain of STEM careers.

The present research has immediate practical applications because the conclusions of this study will allow the IGF to improve the effectiveness of its role-model program. Taking into account the results obtained, the sessions would be enhanced significantly if they were focused especially on the counterstereotypical skills and abilities that are needed to pursue a STEM career, along with information about job opportunities in the new age of automation, the social and practical contributions of STEM fields, and the possibility of achieving work–life balance.

### Practical Implications of the Present Research

The findings from the present study also have practical implications. This study shows the effectiveness of the role-model sessions in terms of reducing gender stereotypes, increasing enjoyment and importance-related values as well as expectation of success, and strengthening the direct effect of expectancies of success on girls’ STEM choices. This research thus demonstrates the benefits of role-model sessions in increasing STEM intention of enrollment among young girls, and thereby suggests a promising method of increasing the number of STEM graduates to meet the growing need for STEM professionals.

An increase in women’s presence within STEM professions is particularly important so as to enable women to seize the new opportunities offered by digital transformation. If women continue to be underrepresented in STEM fields oriented to the design and production of digital technologies, they may fall further behind in the labor market. The World Economic Forum [WEF] (2020) suggests that there is an urgent need to increase the supply and visibility of women with technical skills to close the gender gap in the professions of the future.

In this regard, Madgavkar et al. (2019) estimate that, globally, between 40 million and 160 million women (7–24% of those currently employed) may need to transition between occupations by 2030, often into higher-skilled roles. To make these transitions, women will need new skills. In particular, they will need to overcome their low participation in STEM fields compared to men, as an important barrier that, if not broken, will make it harder for women to make transitions. Policymakers and organizations must step up interventions targeted especially at women, such as addressing stereotypes about occupations and supporting women in STEM professions, which is precisely at the core of the studied role-model sessions.

Although not the main objective of the program, an important positive spillover of these interventions has to do with addressing the issue of vertical sex segregation. This is relevant because, according to the literature, increasing women’s visibility and power in male-dominated occupations will reduce the persistent gender stereotyping, discrimination, and perceptions of lack of belongingness and interest that pose barriers to women’s representation in managerial roles (Gaucher et al., 2011; Skaggs et al., 2012). This potential benefit could come from the fact that many female role models who participate in the sessions are successful professionals who have broken the glass ceiling (i.e., they have been promoted into the upper echelons of their organization). Indeed, some of the strategies that have been posed for fostering greater equality and gender integration in the workplace are focused on the supply side (i.e., women) and include efforts to increase women’s interest in male-typed occupations, such as leadership positions and/or male-dominated STEM fields, through programs targeted at precollege girls to develop their confidence and challenge the cultural contexts that restrict the spectrum of self-beliefs they find acceptable and desirable in gendered ways (Eccles, 1994; Cech et al., 2011; Cech, 2013).

The present research, along with widening the professional horizons of young girls and fostering their interest in male-dominated professions such as STEM careers, shows that these type of intervention could have a positive impact in raising girls’ aspirations by reducing stereotypes about women’s suitability for leadership positions in STEM (Kanter, 1977; Richman et al., 2011; Beasley and Fischer, 2012). Male-dominated STEM careers are frequently associated with decision-making positions (Sáinz et al., 2019).

Nevertheless, other scholars (Seron et al., 2018) claim that promoting greater gender integration alone to effectively raise the low proportion of women in STEM fields is unlikely to achieve cultural change. Indeed, Seron et al. (2018) argue that these types of actions on the supply side would effectively raise the number of women entering STEM careers, but they would not guarantee their persistence in STEM fields, especially in the presence of several structural–cultural factors of women’s marginality, such as the hegemony of the meritocracy and the role of a professional culture that drives token experiences. In this last case, supply-side interventions should be complemented with demand-side actions such as diversity programs by policymakers and companies to ensure that women are equally represented in all phases of the talent pipeline, as recommended by the World Economic Forum [WEF] (2020).

Finally, all these measures should be accompanied by a learning and social environment that promotes the reduction of sexist attitudes and helps to configure a world without stereotypes (Solbes-Canales et al., 2020). Only in this way can the next generation of potential female scientists believe that they can achieve a successful STEM career.

### Limitations and Directions for Future Research

The present results are based on a survey with self-selected schools, and it would be desirable to use a larger sample of schools to reinforce the statistical validity of the results obtained. However, because this is a real and non-laboratory-based study, the design of the sample procedure is beyond the reach of the researcher, who is limited to collecting data in the real environment in which the program is being implemented. Second, and as a consequence of the previous limitation, it could be argued that the results reported in this study are bounded in the sample and might not reflect the patterns of the overall population of young female adolescents in Spain regarding the motivational factors that drive their underrepresentation in STEM fields and the effectiveness of the role-model interventions in these fields. However, the schools that went through the role-model sessions included several regional and socioeconomic varieties, including both public and private schools, giving a relatively diverse sample. Third, the effect of the counterstereotypical content offered by the role models during the sessions in the female adolescents’ career choices suggested by the theoretical model would need to be explored further over a longer period of time, with longitudinal data. This could be carried out through a third-wave survey, at least 3 months after participants attended the role-model sessions, to evaluate their possible residual effect. To do this, it would be necessary to have a larger sample because of the revisable high drop-out rate.

The IGF has developed a new means of exposure to role models through videos, which is easier to implement than face-to-face sessions. It would be interesting for future work to understand which of the two types of intervention is more effective, as the video library has important advantages in terms of cost-effectiveness and time flexibility. The findings from the present study suggest other promising directions for future research. Future work could consider expanding upon the current research with a longitudinal study with repeated exposure to role-model sessions. This would facilitate understanding of the long-lasting effects of role-model exposure. Additionally, because the IGF has started a process of international expansion, mainly in Latin American countries, it could be interesting to evaluate the influence of these role-model sessions across different cultural settings.

Further research should also incorporate a control group of female students who, being in possession of the same features as the final participants, have not been involved in the role-model sessions. This would be key for generalizability, although this has to be done carefully because of ethical concerns about the injustice of omitting a group of girls who could have benefited in the future by attending these role-model sessions. The IGF does not want to discriminate against a group of girls for study reasons. The measurement of STEM choice as a global compendium of different STEM disciplines could be another limitation, this being because the content and objectives of engineering as a discipline (although related) are not the same as those of physical science, computer science, and math. The interest of female students in pursuing physical science could thus be different from their interest in math, computer science, or engineering.

## Data Availability Statement

The raw data supporting the conclusions of this article will be made available by the authors, without undue reservation, to any qualified researcher.

## Ethics Statement

The studies involving human participants were reviewed and approved by Marta Pérez-Dorao (Fundación Inspiring Girls) Carmen Calderón (Universidad CEU San Pablo) Mirian González Durántez (Inspiring Girls Chair). Written informed consent to participate in this study was provided by the participants’ legal guardian/next of kin.

## Author Contributions

RM, SG-P, and MS designed, performed, and analyzed the research, searched literature, and wrote the manuscript. All authors contributed to the article and approved the submitted version.

## Conflict of Interest

The authors declare that the research was conducted in the absence of any commercial or financial relationships that could be construed as a potential conflict of interest.

## References

[B1] Anderson L. S. Gilbride K. A. (2003). Pre-university outreach: encouraging students to consider engineering careers. *Global J. Eng. Educ.* 7 87–93.

[B2] Barbercheck M. (2001). “Mixed messages: men and women in advertisements in science,” in *Women, Science, and Technology: A Reader in Feminist Science Studies*, eds Wyer M. Barbercheck M. Cookmeyer D. Ozturk H. Wayne M., (London: Routledge), 117–131.

[B3] Barker V. L. III Mueller G. C. (2002). CEO characteristics and firm R&D spending. *Manage. Sci.* 48 782–801. 10.1287/mnsc.48.6.782.187 19642375

[B4] Beasley M. A. Fischer M. J. (2012). Why they leave: the impact of stereotype threat on the attrition of women and minorities from science, math and engineering majors. *Soc. Psychol. Educ.* 15 427–448. 10.1007/s11218-012-9185-3

[B5] Bentler P. M. (1995). *EQS Structural Equations Program Manual*, Vol. 6 Encino, CA: Multivariate software.

[B6] Betz D. E. Sekaquaptewa D. (2012). My fair physicist? Feminine math and science role models demotivate young girls. *Soc. Psychol. Pers. Sci.* 3 738–746. 10.1177/1948550612440735

[B7] Breda T. Grenet J. Monnet M. Effenterre C. (2018). “Can female role models reduce the gender gap in science? Evidence from classroom interventions in French high schools,” in PSE Working Papers halshs-01713068 Paris: HAL.

[B8] Bertrand M. Duflo E. (2017). “Field experiments on discrimination,” in *Handbook of Economic Field Experiments*, Vol. 1 eds Banerjee A. V. Duflo E. (North-Holland: National Bureau of Economic Research, Inc), 309–393. 10.1016/bs.hefe.2016.08.004

[B9] Bussey K. Bandura A. (1999). Social cognitive theory of gender development and differentiation. *Psychol. Rev.* 106 676–713. 10.1037/0033-295X.106.4.676 10560326

[B10] Byrne B. M. (1998). *Structural Equation Modeling: Basic Concepts, Application, and Programming.* Mahwah, NJ: Lawrence Earlbaum.

[B11] Byrne B. M. (2010). *Structural Equation Modeling with Amos: Basic Concepts, Applications, and Programming*, 2nd Edn New York, NY: Taylor and Francis Group, 10.12691/education-4-2-10

[B12] Campbell D. T. Stanley J. C. (1963). “Experimental and quasi-experimental designs for research on teaching,” in *Handbook of Research on Teaching*, ed. Gage N. L., (Chicago, IL: Rand McNally), 171–246.

[B13] Cardador M. T. (2017). Promoted up but also out? The unintended consequences of increasing women’s representation in managerial roles in engineering. *Organ. Sci.* 28 597–617. 10.1287/orsc.2017.1132 19642375

[B14] Casad B. Oyler D. L. Sullivan E. T. McClellan E. M. Tierney D. N. Anderson D. A. (2018). Wise psychological interventions to improve gender and racial equality in STEM. *Group Process. Intergroup Relat.* 21 767–787. 10.1177/1368430218767034

[B15] Cech E. Rubineau B. Silbey S. Seron C. (2011). Professional role confidence and gendered persistence in engineering. *Am. Soc. Rev.* 76 641–666. 10.1177/0003122411420815

[B16] Cech E. A. (2013). The self-expressive edge of occupational sex segregation. *Am. J. Soc.* 119 747–789. 10.1086/673969

[B17] Cheryan S. Master A. Meltzoff A. N. (2015). Cultural stereotypes as gatekeepers: increasing girls’ interest in computer science and engineering by diversifying stereotypes. *Front. Psychol.* 6:49. 10.3389/fpsyg.2015.00049 25717308 PMC4323745

[B18] Cheryan S. Siy J. O. Vichayapai M. Drury B. J. Kim S. (2011). Do female and male role models who embody STEM stereotypes hinder women’s anticipated success in STEM? *Soc. Psychol. Pers. Sci.* 2 656–664. 10.1177/1948550611405218

[B19] Correll S. J. (2001). Gender and the career choice process: the role of biased self-assessments. *Am. J. Sociol.* 106 1691–1730. 10.1086/321299

[B20] Diekman A. B. Eagly A. H. (2008). “Of men, women, and motivation,” in *Handbook of Motivation Science*, eds Shah J. Y. Gardner W. L., (New York, NY: Guilford Press), 434–447.

[B21] Diekman A. B. Brown E. R. Johnston A. M. Clark E. K. (2010). Seeking congruity between goals and roles: a new look at why women opt out of science, technology, engineering, and mathematics careers. *Psychol. Sci.* 21 1051–1057. 10.1177/0956797610377342 20631322

[B22] Dimitrov D. M. Rumrill P. D. Jr. (2003). Pretest-posttest designs and measurement of change. *Work* 20 159–165. 12671209

[B23] Dasgupta N. (2011). Ingroup experts and peers as social vaccines who inoculate the self-concept: the stereotype inoculation model. *Psychol. Inquiry* 22 231–246. 10.1080/1047840X.2011.607313

[B24] Durik A. M. Vida M. Eccles J. S. (2006). Task values and ability beliefs as predictors of high school literacy choices: a developmental analysis. *J. Educ. Psychol.* 98:382 10.1037/0022-0663.98.2.382

[B25] Eagly A. H. Wood W. (2011). “Social role theory,” in *Handbook of Theories in Social Psychology*, Vol. 2 eds van Lange P. Kruglanski A. Higgins E. T., (Thousand Oaks, CA: Sage Publications), 458–476. 10.4135/9781446201022.n49

[B26] Eccles J. S. (1987). Gender roles and women’s achievement-related decisions. *Psychol. Women Q.* 11 135–172. 10.1111/j.1471-6402.1987.tb00781.x

[B27] Eccles J. S. (1994). Understanding women’s educational and occupational choices: applying the Eccles et al. model of achievement-related choices. *Psychol. Women Quarterly* 18 585–609. 10.1111/j.1471-6402.1994.tb01049.x

[B28] Eccles J. S. (2005). “Subjective task value and the Eccles et al. model of achievement-related choices,” in *Handbook of Competence and Motivation*, eds Elliot A. J. Dweck C. S., (New York, NY: Guilford Press), 105–121.

[B29] Eccles J. S. (2008). *Can Middle School Reform Increase High School Graduation Rates?* Santa Barbara, CA: University of California.

[B30] Eccles J. S. (2009). Who am I and what am I going to do with my life? Personal and collective identities as motivators of action. *Educ. Psychol.* 44 78–89. 10.1080/00461520902832368

[B31] Eccles J. S. (2015). Gendered socialization of STEM interests in the family. *J. Gender Sci. Technol.* 7 117–132.

[B32] Eccles J. S. Harold R. D. (1991). Gender differences in sport involvement: applying the eccles’ expectancy-value model. *J. Appl. Sport Psychol.* 3 7–35. 10.1080/10413209108406432

[B33] Eccles J. S. Wigfield A. (1995). In the mind of the actor: the structure of adolescents’ achievement task values and expectancy-related beliefs. *Pers. Soc. Psychol. Bull.* 21 215–225. 10.1177/0146167295213003

[B34] Eccles J. S. Wigfield A. Schiefele U. (1998). “Motivation to succeed,” in *(1998). Handbook of Child Psychology: Social, Emotional, and Personality Development*, eds Damon W. Eisenberg N., (Hoboken, NJ: John Wiley & Sons), 1017–1095.

[B35] Eccles J. S. Adler T. F. Futterman R. Goff S. B. Kaczala C. M. Meece J. L. (1983). “Expectancies, values and academic behaviors,” in *Achievement and Achievement Motivation*, ed. Spence J. T., (San Francisco, CA: Freeman), 75–146.

[B36] Enders C. K. (2010). *Applied Missing Data Analysis.* New York, NY: Guilford Press.

[B37] Fornell C. Larcker D. F. (1981). Evaluating structural equation models with unobservable variables and measurement error. *J. Market. Res.* 18 39–50. 10.2307/3151312

[B38] Frenzel A. C. Pekrun R. Goetz T. (2007). Girls and mathematics – A “hopeless” issue? A control-value approach to gender differences in emotions towards mathematics. *Eur. J. Psychol. Educ.* 22 497–514. 10.1007/BF03173468

[B39] Gaucher D. Friesen J. Kay A. C. (2011). Evidence that gendered wording in job advertisements exists and sustains gender inequality. *J. Pers. Soc. Psychol.* 101:109. 10.1037/a0022530 21381851

[B40] Glick P. Fiske S. T. (1999). “Sexism and other “isms”: independence, status, and the ambivalent content of stereotypes,” in *Sexism and Stereotypes in Modern Society: The Gender Science of Janet Taylor Spence*, eds Swann W. B. Langlois J. H. Gilbert L. A., (Washington, DC: American Psychological Association), 193–221. 10.1037/10277-008

[B41] Good C. Rattan A. Dweck C. S. (2012). Why do women opt out? Sense of belonging and women’s representation in mathematics. *J. Pers. Soc. Psychol.* 102 700–717. 10.1037/a0026659 22288527

[B42] Guimond S. Roussel L. (2001). Bragging about one’s school grades: gender stereotyping and students’ perception of their abilities in science, mathematics, and language. *Soc. Psychol. Educ.* 4 275–293. 10.1023/A:1011332704215

[B43] Hackett G. Betz N. E. (1981). A self-efficacy approach to the career development of women. *J. Vocat. Behav.* 18 326–339. 10.1016/0001-8791(81)90019-1

[B44] Hair J. F. Ringle C. M. Sarstedt M. (2011). PLS-SEM: indeed a silver bullet. *J. Market. Theory Pract.* 19 139–152. 10.2753/MTP1069-6679190202

[B45] Hitt M. A. Tyler B. B. (1991). Strategic decision models: integrating different perspectives. *Strategic Manage. J.* 12 327–351. 10.1002/smj.4250120502

[B46] Hu L. Bentler P. M. (1999). Cutoff criteria for fit indexes in covariance structure analysis: conventional criteria versus new alternatives. *Struct. Equation Model.* 6 1–55. 10.1080/10705519909540118

[B47] Inspiring Girls Foundation [IGF], (2018). *Inspiring Girls Foundation Annual Review.* London: IGF.

[B48] Johnson C. C. Mohr-Schroeder M. J. Moore T. J. English L. D. (2020). *Handbook of Research on STEM Education.* New York, NY: Routledge.

[B49] Kahn S. Ginther D. (2017). *Women and STEM.* NBER Working Papers 23525, Cambridge, MA: National Bureau of Economic Research, Inc.

[B50] Kang H. Calabrese Barton A. Tan E. Simpkins S. D. Rhee H. Y. Turner C. (2019). How do middle school girls of color develop STEM identities? Middle school girls’ participation in science activities and identification with STEM careers. *Sci. Educ.* 103 418–439. 10.1002/sce.21492

[B51] Kanter R. M. (1977). Some effects of proportions on group life: skewed sex ratios and responses to token women. *Am. J. Sociol.* 82 965–990. 10.1086/226425

[B52] Kesar S. (2018). *Closing the STEM gap: Why STEM Classes and Careers Still Lack of Girls and What Can we do About it. Microsoft.* Available online at: https://htmlglobal.com/closing-the-stem-gap-why-stem-classes-and-careers-still-lack-girls-and-what-we-can-do-about-it/ (accessed June 30, 2020).

[B53] Knapp T. (2016). Why is the one-group pretest-posttest design still used? *Clin. Nurs. Res.* 25 467–472. 10.1177/1054773816666280 27558917

[B54] Langdon D. McKittrick G. Beede D. Khan B. Doms M. (2011). *STEM: Good Jobs Now and for the Future.* Washington, DC: U.S. Department of Commerce.

[B55] Li Q. (2007). Mathematics, science, and technology in secondary schools: do gender and region make a difference? *Can. J. Learn. Technol.* 33 41–57. 10.21432/T2N018

[B56] Little T. D. (2013). *Longitudinal Structural Equation Modeling.* New York, NY: Guildford Press.

[B57] Lockwood P. (2006). Someone like me can be successful”: do college students need same-gender role models? *Psychol. Women Quarterly* 30 36–46. 10.1111/j.1471-6402.2006.00260.x

[B58] Lockwood P. Kunda Z. (1997). Superstars and me: predicting the impact of role models on the self. *J. Pers. Soc. Psychol.* 73 91–103. 10.1037/0022-3514.73.1.91

[B59] London B. Rosenthal L. Levy S. R. Lobel M. (2011). The influences of perceived identity compatibility and social support on women in nontraditional fields during the college transition. *Basic Appl. Soc. Psychol.* 33 304–321. 10.1080/01973533.2011.614166

[B60] Madgavkar A. Manyika J. Krishnan M. Ellingrud K. Yee L. Woetzel J. (2019). *The Future of Women at Work: Transitions in the Age of Automation.* Philadelphia, PA: McKinsey & Co.

[B61] Martin C. L. Halverson C. F. Jr. (1981). A schematic processing model of sex typing and stereotyping in children. *Child Dev*. 52 1119–1134. 10.2307/1129498

[B62] Marx D. M. Stapel D. A. Muller D. (2005). We can do it: the interplay of construal orientation and social comparisons under threat. *J. Pers. Soc. Psychol.* 88 432–446. 10.1037/0022-3514.88.3.432 15740438

[B63] Ministerio de Educación y Formación Profesional (MEFP) (2019). *Enseñanzas Universitarias. Alumnado matriculado. [Ministry of Education and Vocational Training Non-university education]. University enrollments].* Available online at: www.educacionyfp.gob.es/servicios-al-ciudadano/estadisticas/universitaria/estadisticas.html (accessed December 10, 2019).

[B64] Ministerio de Educación y Formación Profesional (MEFP) (2020). *Enseñanzas Universitarias. Alumnado matriculado. [Ministry of Education and Vocational Training Non-university education]. University enrollments].* Available online at: www.educacionyfp.gob.es/servicios-al-ciudadano/estadisticas/universitaria/estadisticas.html (accessed June 26, 2020).

[B65] Nunnally J. C. Bernstein I. (1994). *Psychometric Theory*, 3rd Edn New York, NY: McGraw-Hill 10.1177/014662169501900308

[B66] O’Brien L. T. Hitti A. Shaffer E. Camp A. R. V. Henry D. Gilbert P. N. (2017). Improving girls’ sense of fit in science: increasing the impact of role models. *Soc. Psychol. Pers. Sci.* 8 301–309. 10.1177/1948550616671997

[B67] Olsson M. Martiny S. E. (2018). Does exposure to counterstereotypical role models influence Girls’ and Women’s gender stereotypes and career choices? A review of social psychological research. *Front. Psychol.* 9:2264. 10.3389/fpsyg.2018.02264 30581398 PMC6292925

[B68] Organization for Economic Co-operation and Development (OECD), (2018a). *Distribution of Graduates and Entrants by Field: Share of Graduates by Gender and by Field.* Available online at: stats.oecd.org/index.aspx?queryid=79486# (accessed November 11, 2019).

[B69] Organization for Economic Co-operation and Development (OECD), (2018b). *Employment Labor Participation Rate.* Available online at: stats.oecd.org/index.aspx?queryid=54741 (accessed November 11, 2019).

[B70] Organization for Economic Co-operation and Development (OECD), (2018c). *Indicator B5: Who is Expected to Graduate from Tertiary Education? In Education at a Glance 2018: OECD Indicators.* Paris: OECD Publishing.

[B71] Plant E. Ashby, Baylor A. L. Doerr C. E. Rosenberg-Kima R. B. (2009). Changing middle-school students’ attitudes and performance regarding engineering with computer-based social models. *Comp. Educ.* 53 209–215. 10.1016/j.compedu.2009.01.013

[B72] Quimby J. L. O’Brien K. M. (2004). Predictors of student and career decision-making self-efficacy among nontraditional college women. *Career Dev. Quarterly* 52 323–339. 10.1002/j.2161-0045.2004.tb00949.x

[B73] Raykov T. Marcoulides G. A. (2000). *A First Course in Structural Equation Modeling.* Mahwah, NJ: Lawrence Earlbaum Associates 10.1080/10705510701758448

[B74] Richman L. S. Vandellen M. Wood W. (2011). How women cope: being a numerical minority in a male-dominated profession. *J. Soc. Issues* 67 492–509. 10.1111/j.1540-4560.2011.01711.x

[B75] Rigdon E. Schumacker R. E. Wothke W. (1998). “A comparative review of interaction and nonlinear modeling,” in *Interaction and Nonlinear Effects in Structural Equation Modeling*, eds Schumacker R. E. Marcoulides G. A., (Mahwah, NJ: Lawrence Erlbaum).

[B76] Rosenthal L. London B. Levy S. R. Lobel M. (2011). The roles of perceived identity compatibility and social support for women in a single-sex STEM program at a co-educational university. *Sex Roles* 65 725–736. 10.1007/s11199-011-9945-0

[B77] Ruigrok W. Peck S. Tacheva S. (2007). Nationality and gender diversity on Swiss corporate boards. *Corporate Govern. Int. Rev.* 15 546–557. 10.1111/j.1467-8683.2007.00587.x

[B78] Sáinz M. (2020). *Brechas y Sesgos de Género en la Elección de Estudios stem. por qué Ocurren y cómo Actuar Para Eliminarlas?* [Gender Gaps and Biases in the Choice of STEM. Why They Occur and How to Act to Eradicate them?]. Sevilla: Centro de Estudios Andaluces.

[B79] Sáinz M. Eccles J. (2012). Self-concept of computer and math ability: gender implications across time and within ICT studies. *J. Vocat. Behav.* 80 486–499. 10.1016/j.jvb.2011.08.005

[B80] Sáinz M. Martínez-Cantos J. L. Rodó-de-Zárate M. Romano M. J. Arroyo L. Fàbregues S. (2019). Young Spanish people’s gendered representations of people working in STEM. A qualitative study. *Front. Psychol.* 10:996. 10.3389/fpsyg.2019.00996 31133933 PMC6514192

[B81] Sáinz M. Meneses J. López B. Fàbregues S. (2016). Gender stereotypes and attitudes towards ICT in a sample of Spanish secondary students. *Sex Roles* 74 154–168. 10.1007/s11199-014-0424-2

[B82] Seron C. Silbey S. S. Cech E. Rubineau B. (2016). Persistence is cultural: professional socialization and the reproduction of sex segregation. *Work Occup.* 43 178–214. 10.1177/0730888415618728

[B83] Seron C. Silbey S. Cech E. Rubineau B. (2018). “I am not a feminist, but.”: hegemony of a meritocratic ideology and the limits of critique among women in engineering. *Work Occup.* 45 131–167. 10.1177/0730888418759774

[B84] Shapiro J. R. Baldwin M. Williams A. M. Trawalter S. (2011). The company you keep: fear of rejection in intergroup interaction. *J. Exp. Soc. Psychol.* 47 221–227. 10.1016/j.jesp.2010.10.006

[B85] Shin J. E. L. Levy S. R. London B. (2016). Effects of role model exposure on STEM and non-STEM student engagement. *J. Appl. Soc. Psychol.* 46 410–427. 10.1111/jasp.12371

[B86] Skaggs S. Stainback K. Duncan P. (2012). Shaking things up or business as usual? The influence of female corporate executives and board of directors on women’s managerial representation. *Soc. Sci. Res.* 41 936–948. 10.1016/j.ssresearch.2012.01.006 23017861

[B87] Solbes-Canales I. Valverde-Montesino S. Herranz-Hernández P. (2020). Socialization of gender stereotypes related to attributes and professions among young Spanish school-aged children. *Front. Psychol.* 11:609. 10.3389/fpsyg.2020.00609 32390895 PMC7194082

[B88] Starr C. R. (2019). *That’s Not Me”: STEM Stereotypes, Self-Concepts, and Motivation.* Doctoral dissertation, UC Santa Cruz, Santa Cruz, CA.

[B89] Stoeger H. Schirner S. Laemmle L. Obergriesser S. Heilemann M. Ziegler A. (2016). A contextual perspective on talented female participants and their development in extracurricular STEM programs. *Ann. New York Acad. Sci.* 1377 53–66. 10.1111/nyas.13116 27442498

[B90] Stout J. G. Dasgupta N. Hunsinger M. McManus M. A. (2011). STEMing the tide: using ingroup experts to inoculate women’s self-concept in science, technology, engineering, and mathematics (STEM). *J. Pers. Soc. Psychol.* 100 255–270. 10.1037/a0021385 21142376

[B91] Thébaud S. Charles M. (2018). Segregation, stereotypes, and STEM. *Soc. Sci.* 7:111 10.3390/socsci7070111

[B92] Van Camp A. R. Gilbert P. N. O’Brien L. (2019). Testing the effects of a role model intervention on women’s STEM outcomes. *Soc. Psychol. Educ.* 22 649–671. 10.1007/s11218-019-09498-2

[B93] Walton G. M. Cohen G. L. (2007). A question of belonging: race, social fit, and achievement. *J. Pers. Soc. Psychol.* 92 82–96. 10.1037/0022-3514.92.1.82 17201544

[B94] Wang M. T. Degol J. L. (2014). Motivational pathways to STEM career choices: using expectancy-value perspective to understand individual and gender differences in STEM fields. *Dev. Rev.* 33 304–340. 10.1016/j.dr.2013.08.001 24298199 PMC3843492

[B95] Wang M. T. Degol J. Ye F. (2015). Math achievement is important, but task values are critical, too: examining the intellectual and motivational factors leading to gender disparities in STEM careers. *Front. Psychol.* 6:36. 10.3389/fpsyg.2015.00036 25741292 PMC4330678

[B96] Wang M. T. Ye Y. Degol J. L. (2017). Who chooses STEM careers? Using a relative cognitive strength and interest model to predict careers in science, technology, engineering, and mathematics. *J. Youth Adolescence* 46 1805–1820. 10.1007/s10964-016-0618-8 27975183

[B97] Watt H. M. Shapka J. D. Morris Z. A. Durik A. M. Keating D. P. Eccles J. S. (2012). Gendered motivational processes affecting high school mathematics participation, educational aspirations, and career plans: a comparison of samples from Australia, Canada, and the United States. *Dev. Psychol.* 48:1594. 10.1037/a0027838 22468566

[B98] Weisgram E. S. Bigler R. S. (2007). Effects of learning about gender discrimination on adolescent girls’ attitudes toward and interest in science. *Psychol. Women Quarterly* 31 262–269. 10.1111/j.1471-6402.2007.00369.x

[B99] Wigfield A. Eccles J. S. (1992). The development of achievement task values: a theoretical analysis. *Dev. Rev.* 12 265–310. 10.1016/0273-2297(92)90011-P

[B100] Wigfield A. Eccles J. S. (2000). Expectancy–value theory of achievement motivation. *Contemp. Educ. Psychol.* 25 68–81. 10.1006/ceps.1999.1015 10620382

[B101] Wigfield A. Eccles J. S. (eds). (2002). *Development of achievement motivation.* San Diego, CA: Elsevier.

[B102] Wigfield A. Eccles J. S. Schiefele U. Roeser R. W. Davis-Kean P. (2006). “Social, emotional, and personality development,” in *Handbook of Child Psychology*, 6th Edn, ed. Eisenberg N., (Hoboken, NJ: Wiley), 933–1002.

[B103] Williams W. M. Ceci S. J. (2012). When scientists choose motherhood: a single factor goes a long way in explaining the dearth of women in math-intensive fields. How can we address it? *Am. Sci.* 100 138–145. 10.1511/2012.95.138 24596430 PMC3939045

[B104] Willms J. D. Jacobsen S. (1990). Growth in mathematics skills during the intermediate years: sex differences and school effects. *Int. J. Educ. Res.* 14 157–174.

[B105] World Economic Forum [WEF], (2020). *Global Gender Gap Report.* Geneva: World Economic Forum.

